# Capacity Limits Lead to Information Bottlenecks in Ongoing Rapid Motor Behaviors

**DOI:** 10.1523/ENEURO.0289-22.2023

**Published:** 2023-03-13

**Authors:** Richard Hugh Moulton, Karen Rudie, Sean P. Dukelow, Brian W. Benson, Stephen H. Scott

**Affiliations:** 1 Department of Electrical and Computer Engineering, Queen’s University, Kingston, Ontario, ON K7L 3N6, Canada; 2 School of Computing, Queen’s University, Kingston, Ontario, ON K7L 2N8, Canada; 3 Ingenuity Labs Research Institute, Queen’s University, Kingston, Ontario, ON K7L 3N6, Canada; 4 Department of Clinical Neurosciences, University of Calgary, Calgary, Alberta, AB T2N 1N4, Canada; 5 Hotchkiss Brain Institute, University of Calgary, Calgary, Alberta, AB T2N 1N4, Canada; 6 Cumming School of Medicine, University of Calgary, Calgary, Alberta, AB T2N 1N4, Canada; 7 Benson Concussion Institute, Calgary, Alberta, AB T3B 6B7, Canada; 8 Canadian Sport Institute Calgary, Calgary, Alberta, AB T3B 5R5, Canada; 9 Department of Biomedical and Molecular Sciences, Queen’s University, Kingston, Ontario, ON K7L 3N6, Canada; 10 Centre for Neuroscience Studies, Queen’s University, Kingston, Ontario, ON K7L 3N6, Canada; 11 Department of Medicine, Queen’s University, Kingston, Ontario, ON K7L 3N6, Canada

**Keywords:** attention, human action, perceptual decision-making, Rapid motor behavior, reaching movements

## Abstract

Studies of ongoing, rapid motor behaviors have often focused on the decision-making implicit in the task. Here, we instead study how decision-making integrates with the perceptual and motor systems and propose a framework of limited-capacity, pipelined processing with flexible resources to understand rapid motor behaviors. Results from three experiments show that human performance is consistent with our framework: participants perform objectively worse as task difficulty increases, and, surprisingly, this drop in performance is largest for the most skilled performers. As well, our analysis shows that the worst-performing participants can perform equally well under increased task demands, which is consistent with flexible neural resources being allocated to reduce bottleneck effects and improve overall performance. We conclude that capacity limits lead to information bottlenecks and that processes like attention help reduce the effects that these bottlenecks have on maximal performance.

## Significance Statement

Behavior results from perception, decision-making, and motor control. These processes can be modelled serially, but we must often decide about future actions in parallel with ongoing actions. We use computational models to demonstrate that simply perceiving, deciding, and moving in parallel inevitably produces performance-limiting interference. We see that the best performers in rapid motor behavior tasks are counter-intuitively the most affected by increasing task demands, which is consistent with sharing attention between pipelined processes.

## Introduction

Reaction time tasks are a classic way to explore perceptual, decision-making, and motor processes, as response times reflect the summation of these processes. Response times can be as fast as 100 ms for a preplanned motor response (Scott, 2016) or as long as 700 ms when identifying emotions from a person’s face (Nook et al., 2015). This “decide-then-act” paradigm has supplied a number of experiments used to study decision-making during motor actions, including motor lotteries (Trommershäuser et al., 2008; Wu et al., 2009; Nagengast et al., 2011; Jarvstad et al., 2013; Neyedli and Welsh, 2013, 2015; Neyedli and LeBlanc, 2017; Adkins et al., 2022), reaching tasks (Gallivan et al., 2011, 2015; Cos et al., 2014; Enachescu et al., 2021; Ulbrich and Gail, 2021), foraging tasks (Diamond et al., 2017), and intercept tasks (Barany et al., 2020; Fooken and Spering, 2020; Michalski et al., 2020).

Less is known about how perceptual, decision-making, and motor processes interact to generate behavior. Even in the “decide-then-act” paradigm, models such as the affordance competition hypothesis (Cisek, 2007) and embodied decision-making (Cisek and Pastor-Bernier, 2014; Lepora and Pezzulo, 2015) argue that these processes work in parallel, but the situation becomes much more complex when we must “decide-while-acting” (Michalski et al., 2020). Sight-reading piano music, for example, requires the pianist to perceive notes on the sheet music and decide how to play subsequent key strokes while actively playing the musical piece. Future actions are planned while current actions are executed, making future actions a component of the present decision (Gallivan et al., 2018), and providing an opportunity for parallel processing and feedback between processes (Lepora and Pezzulo, 2015). Cisek and Kalaska argued that the parallel characteristics of motor decision-making in humans evolved in response to an environment that requires ongoing, interactive behavior (Cisek and Kalaska, 2010). Parallel processing allows individuals to respond to a stimulus faster when it is the second in a sequence compared with being presented on its own, especially when the two stimuli are compatible, but it breaks down under difficult speeded-response conditions and serial processing is forced (Kahneman, 1973). We ask the question, at what point do the brain’s time-sensitive processing capabilities limit the performance of rapid motor behaviors?

One important consideration is that perceptual, decision-making, and motor processes interact and interfere with one another (Passingham, 1996; Scherbaum et al., 2015; Künstler et al., 2018; Carroll et al., 2019; Thura, 2020; Raßbach et al., 2021; Ulbrich and Gail, 2021). This interference effect has been explained by models that posit either a structural bottleneck or a shared pool of central resources that restrict the brain’s processing capacity (Navon and Miller, 2002). Structural bottleneck models propose that a particular process, such as stimulus perception (Broadbent, 1957) or response selection (Deutsch and Deutsch, 1963), has a fixed capacity. This particular process is the bottleneck and it limits performance. Shared resource models posit instead that the brain must allocate resources, such as attention (Kahneman, 1973) or cognitive maps (Franconeri et al., 2013), between possible activities. For these models, the effects of task characteristics and limited resources can result in any cognitive process becoming the bottleneck.

In this paper, we introduce an information-processing framework with shared resources to interpret how humans produce rapid motor behaviors. We explore how the limited-capacity and pipeline characteristics of this framework lead to information bottlenecks that dictate system performance. We evaluate three models in this framework against datasets of human participants hitting moving objects as part of time-constrained motor behavior tasks in a virtual workspace using an interactive robotic platform (Singh and Scott, 2003). The limited-capacity, pipelined framework correctly predicts that changes in both task demands (e.g., more and faster objects in the workspace) and behavioral demands (e.g., distractor objects adding arbitrary decision rules) lead to complex changes in performance, including that highly skilled individuals tend to be more impacted than those with less skill. These results highlight how changes in a behavioral task may or may not create a bottleneck in performance that partially reflects the individual’s preexisting level of performance.

## Materials and Methods

### Participants

A total of 2303 human participants (1564 males and 738 females, aged 8–93, 2084 right-handed and 195 left-handed) have performed tasks in the Object-Hit (OH) family (Table 1). Of these, 985 participants were recruited through the “Safe to Play” longitudinal research program.

**Table 1:** Participant demographics for rapid motor behavior tasks

	OH	OHA	TOH	TOHA	All Tasks
*n*	618	515	1698	1660	2303
Male	264	225	1306	1289	1564
Female	354	290	391	370	738
Right-handed	553	458	1541	1507	2084
Left-handed	63	55	134	131	195
Ambidextrous	2	2	23	22	24
Age	46 (18, 93)	46 (18, 93)	15 (8, 71)	15 (8, 55)	16 (8, 93)

Number of participants and breakdowns by sex and by handedness are all reported as counts. Breakdowns by sex and handedness may not sum to the total number of participants because of individuals reporting their sex as neither male nor female or individuals being assessed as ambidextrous. Age is reported as median (minimum, maximum).

Participants were recruited from the communities of Kingston, Ontario and Calgary, Alberta. Participants in Calgary, Alberta were recruited through and tested at the Benson Concussion Institute, Group23 Sports Medicine Clinic (formerly WinSport Medicine Clinic). Test procedures were approved by the Research Ethics Boards of Queen’s University, Providence Care, and the University of Calgary. All participants gave their written and informed consent to have their data collected for research purposes. This written and informed consent was obtained from the individual’s parents/guardians if the individual was under the age of 17.

Healthy human participants had no significant neurologic impairments, were medically stable, had normal or corrected to normal visual acuity, had no ongoing musculoskeletal injuries of the upper limb, and were able to understand task instructions (Tyryshkin et al., 2014; Bourke et al., 2016). All participants who performed the turbo variants of the task had their visual acuity confirmed to be 20/40 uncorrected or better (Mang et al., 2018).

For all three experiments, we compared trial performances for participants who have completed both of the two tasks being compared. This resulted in 487 participants for Experiment 1 (212 male, 432 right-handed, age range 18–93), 1662 participants for Experiment 2 (1290 male, 1510 right-handed, age range 8–55), and 38 participants for Experiment 3 (17 male, 34 right-handed, age range 19–71).

### Tasks

The four tasks used in this experimental design have been validated as being suitable for measuring rapid motor behaviors in healthy human participants, as producing reliable test results, and as provoking no learning effects between trials (Mang et al., 2018; Simmatis et al., 2020; Moulton et al., 2022). Although when we compare tasks we might say that one task increases the demands on a particular process, we note that all four tasks require perception, decision-making, and motor control to occur in some proportion. None of these tasks, OH in particular, is made easier by the absence of demands for one of these processes.

#### Object-hit

**Figure 1.**
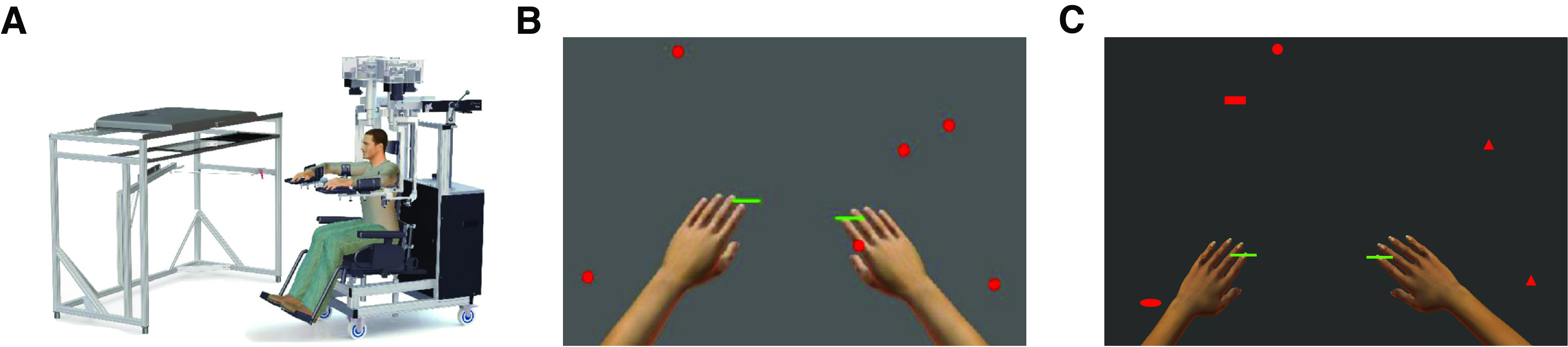
The Object-Hit and Object-Hit-and-Avoid tasks. ***A***, Kinarm Exoskeleton robot, used for the OH and OHA tasks. ***B***, Sample screen shot from the OH task. Objects are dropped in 10 evenly spaced horizontal bins that are not visible to the participant. Arms are shown only for illustrative purposes, only the green paddles are visible during the task. ***C***, Sample screen shot from the OHA task. Objects have eight possible shapes: two shapes indicate target objects and the other six shapes indicate distractor objects.

The OH task was designed to examine an individual’s ability to generate quick and accurate arm movements throughout the workspace (Tyryshkin et al., 2014). Participants were seated in a bilateral Kinarm exoskeleton lab (Kinarm, Kingston, Ontario; Singh and Scott, 2003; Fig. 1*A*), which provides full gravitational support to the upper limbs while allowing movement in the horizontal plane (Tyryshkin et al., 2014).

Participants were instructed to use their hands, represented visually as 5 cm wide green paddles, to hit virtual red objects (circular, 2-cm diameter) that moved toward them in the horizontal plane. These red objects represent targets and appear from 10 different bins (not visible to the participant), with each bin dropping an object in random order before repeating (Fig. 1*B*). Each bin drops 30 objects over the length of the task, resulting in 300 total targets. Positions and velocities of the hands and objects are recorded with a sampling frequency of 200 Hz and the Kinarm exoskeleton generated a 50-ms force pulse to simulate feedback of contact between paddles and objects. For full details, see the original paper by Tyryshkin et al. (2014).

The OH task challenges participants to produce peak performance. Trials begin with objects created at a slow rate and with slow speeds. During this easy phase, the task is “data-limited” and participants’ performance is limited by the input from the task (Wickens, 1981). Throughout the trial, however, objects are steadily created at a faster rate and with faster speeds, increasing perceptual, decision, and motor processing demands (Vidal et al., 2015), ensuring that participants always finished a trial by being overwhelmed. Once they are overwhelmed, participants’ performance is resource-limited as they trade off speed, accuracy, and effort to maximize their global reward rate (Fitts, 1954; Wickens, 1981; Reynaud et al., 2020). We also saw that participants missed many more peripheral targets when they were overwhelmed (Moulton et al., 2022), potentially maximizing reward rate by minimizing movement between targets and consistent with a preference for less effortful actions (Cos et al., 2014).

#### Object-Hit-and-Avoid

The Object-Hit-and-Avoid task (OHA) is similar to OH, but now the red objects have different shapes. Two of the eight shapes indicated targets to hit while the remaining shapes indicated distractors to avoid (Fig. 1*C*). This introduces an arbitrary decision rule and requires some movements to be inhibited. Participants were again seated in a Kinarm exoskeleton lab to record their performance and instructed to hit as many of the 200 targets as possible without hitting the 100 distractor objects (Bourke et al., 2016).

The rate at which objects appeared on the screen and the speed with which they moved steadily increased throughout the trial in an identical manner to OH. Haptic feedback was provided by the exoskeleton on paddle contact with targets. Distractors passed through the paddle with no haptic feedback to provide an instantaneous reminder that it was a distractor. For full details, see the original paper by Bourke et al. (2016).

#### Turbo Object-Hit and Turbo Object-Hit-and-Avoid

The Turbo Object-Hit (TOH) and Turbo Object-Hit-and-Avoid (TOHA) tasks are speeded versions of the original tasks, with faster moving objects and smaller paddles (2 vs 5 cm). The original intent behind the task was to remove the ceiling effect that was specifically observed when elite athletes performed OH and OHA (Mang et al., 2018).

**Figure 2.**
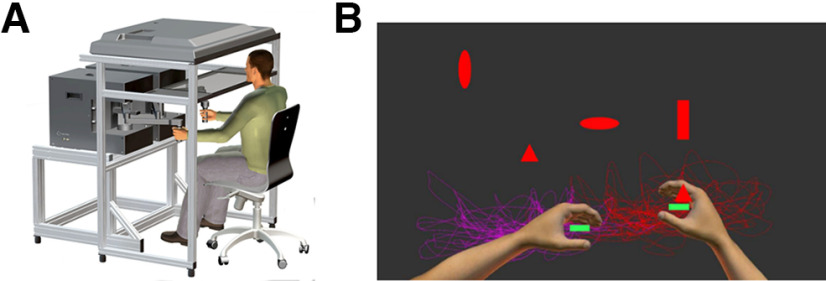
The Turbo tasks. ***A***, Kinarm Endpoint robot, used for the TOH and TOHA tasks. ***B***, Sample screen shot from the TOHA task illustrating the smaller paddle size. Arms and traces, indicating the participant’s movement with the left (purple) and right (red) hands, were not visible during the task.

Participants used a bilateral Kinarm end-point lab (Kinarm, Kingston, Ontario; Fig. 2*A*). Between 2011 and 2013, participants were seated in front of the robot; beginning in 2014 the robot was modified to allow participants to stand. Participants held on to two handles, which moved in the horizontal plane to interact with an augmented reality system displayed in the same plane (Mang et al., 2018).

In all other respects the turbo tasks were the same as the original tasks: TOH has 300 targets, TOHA has 200 targets and 100 distractors, and TOHA chooses two shapes out of eight to indicate these targets every trial. See Mang et al. (2018) for full details.

### Data analysis

Event codes for object creations and object/paddle contacts were extracted from all trial records and object/paddle contacts were assessed as hits or misses depending on the object’s trajectory at termination (consistent with Tyryshkin et al., 2014). These events were converted into instantaneous target creation and target hit rates using two iterations of a Kolmogorov–Zurbenko filter with a sliding window with 5 s width.

The difference between target creation and target hit rates was calculated at each frame and the median difference over a window with 5 s width was calculated. The beginning of a participant’s overwhelmed phase was then determined to be the latest frame at which the participant was overwhelmed, characterized as when the median rate difference, (1), minus the median absolute deviation, (2), was <0.1 Hz:

(1) $$\tilde{X}=\text{median}(X)$$

where *X* is a univariate dataset

(2) $$\text{MAD}(X)=\text{median}(|X_{i}-\tilde{X}|)$$

We characterized participants’ performance during the overwhelmed phase by calculating their steady-state rate: the average target hit rate from the initial frame of the overwhelmed phase to the frame where the trial’s target creation rate peaked. The initial frame for this overwhelmed phase was then brought forward and the steady-state rate recalculated if (1) doing so increased the participant’s steady-state rate or (2) if the median rate difference minus the median absolute deviation was <0.1 Hz at any point in the preceding 5 s. This type of rate parameter is useful for characterizing performance for tasks involving ongoing movement and has been previously used in studying the latency and duration of keystroke sequences for typewriting tasks (Sternberg et al., 1978).

We interpret a participant’s steady-state rate for a trial as representing their peak performance and underlying sensorimotor abilities. Although it is difficult to untangle the effects of factors such as motivation, we believe that this interpretation is justified for three reasons. First, if motivation levels determined steady-state rates within a trial then we would expect to see some participants either substantially increase or substantially decrease their target hit rate during the overwhelmed phase as their motivation fluctuated, but this was not observed. Second, if motivation levels determined steady-state rates between trials then we would expect some participants to exhibit a wide range of steady-state rates across trials. Instead, all four tasks have been validated as evoking consistent performance levels and steady-state rates between tests. Third, if motivation levels determine steady-state rates between participants, this role seems to be consistent both within and between trials for single participants. We would therefore consider this kind of effect as a part of the participant’s underlying ability to perform rapid motor behavior tasks.

### Statistical analysis

Because of the problems associated with frequentist null hypothesis testing (Nuzzo, 2014; Kruschke and Liddell, 2018; Amrhein et al., 2019), we assessed the significance of our results using Bayesian data analysis techniques drawn from Kruschke’s textbook on the subject (Kruschke, 2015)

#### Assessing significant differences

For each experiment we assessed whether or not the difference in steady-state rates was significantly different from 0. For this analysis we assumed that these differences were drawn from a normal distribution with mean *μ* and SD *σ*. The question of interest, then, is whether or not 0 Hz is a credible value for the parameter *μ* (Kruschke, 2015).

Our prior distribution for the parameters was informed by the population-level differences for the tasks. For two arbitrary tasks, our prior belief for *μ* was centered on the difference in average steady-state rates between population with a SD equal to the precision of the baseline task’s steady-state rates; our prior belief for *σ* was a uniform distribution over a wide range of values based on the baseline task’s SD:

$$\begin{array}{l} \mu ∼N(\text{mean}(SSR_{\text{Task 2}})-\text{mean}(SSR_{\text{Task 1}}),\frac{1}{{\left(10\times \sigma_{\text{Task 1}}\right)}^{2}})\\ \sigma ∼U(0.001\times \sigma_{\text{Task 1}},1000\times \sigma_{\text{Task 1}}) \end{array}.$$

We established the region of practical equivalence (ROPE) to capture the range of differences that we will consider as practically equivalent to a difference of 0 Hz. The size of this ROPE was calculated as the intertrial variability of steady-state rates for a single participant; in other words, the degree of variability that we would expect to see from a participant performing the baseline task a second time.

Next, we then calculated the difference in steady-state rates for each participant as our evidence. A Markov chain Monte Carlo (MCMC) method was then used to apply Bayesian inference and calculate our posterior belief regarding the values for parameters *μ* and *σ*. The representativeness of the MCMC chains was assessed by ensuring overlap of the values assumed by each chain, ensuring overlap of the 95% high-density interval (HDI) from each chain, and verifying convergence of the chains by computing the Brooks-Gelman-Rubin statistic (or shrink factor). The accuracy of the MCMC chains was assessed by ensuring that the chains were not autocorrelated over a series of lags, by computing the effective sample size of the four chains, and by considering the Monte Carlo standard error of the chains (Kruschke, 2015).

Finally, we described our posterior belief for these parameter values by reporting the mode parameter value as well as the 95% HDI of the posterior distribution. The 95% HDI for the parameter *μ* was then compared against the ROPE around 0 Hz to determine whether or not participants’ difference in steady-state rates between the two tasks are different enough from 0 Hz for practical purposes.

#### Bayesian linear regression

A similar analysis was performed for each experiment when we conducted a Bayesian linear regression to relate an participant’s steady-state rate in Task 2 with their steady-state rate in Task 1 (Kruschke, 2015). For this analysis, we assumed that steady-state rates for Task 2 were drawn from a *t*-distribution with centrality parameter *μ*, scale parameter *σ*, and normality parameter *ν*. We further assumed that the centrality parameter for any given participant’s distribution was linearly related to their steady-state rate from Task 1:

$$\begin{array}{l} SSR_{\text{Task 2}}∼T_{\nu }(\mu ,\sigma )\\ \mu =\beta_{0}+\beta_{1}\times SSR_{\text{Task 1}} \end{array}.$$

Our prior beliefs for these parameters were:

$$\begin{array}{l} \sigma ∼U(0.001,1000)\\ \nu ∼\text{Exponential}(0.0\bar{333})\\ \beta_{0}∼N(0,0.01)\\ \beta_{1}∼N(1,0.01) \end{array}.$$

For the linear regression parameters, these prior beliefs implied that participants’ steady-state rates would be roughly equal between the two tasks.

We verified for each regression that the estimated values for *σ* and *ν* implied that a linear model was plausible for relating steady-state rates between tasks. The relevant parameters for distinguishing between the three different models, however, were *β*_0_ and *β*_1_. As the y-intercept parameter, we were interested in determining whether or not *β*_0_ was sufficiently different from 0 Hz for practical purposes. We established the ROPE for this value as (–0.12, 0.12), which represented the range of y-intercepts estimated for the Multiplicative Workload systems from our simulations. As the slope parameter, we wanted to determine whether or not *β*_1_ was sufficiently different from 1 for practical purposes. The ROPE for this value was (0.95, 1.05) representing the range of slopes estimated for the Additive Workload systems from our simulations.

An MCMC method was then used to apply Bayesian inference and produce estimates for all four parameters. We described our resulting posterior beliefs for these parameter values by again reporting the mode value and the 95% HDI of the posterior distribution. The 95% HDIs for the parameters *β*_0_ and *β*_1_ were then compared against their respective ROPEs to assess whether or not they reflected a practical difference.

### Simulations

**Figure 3.**
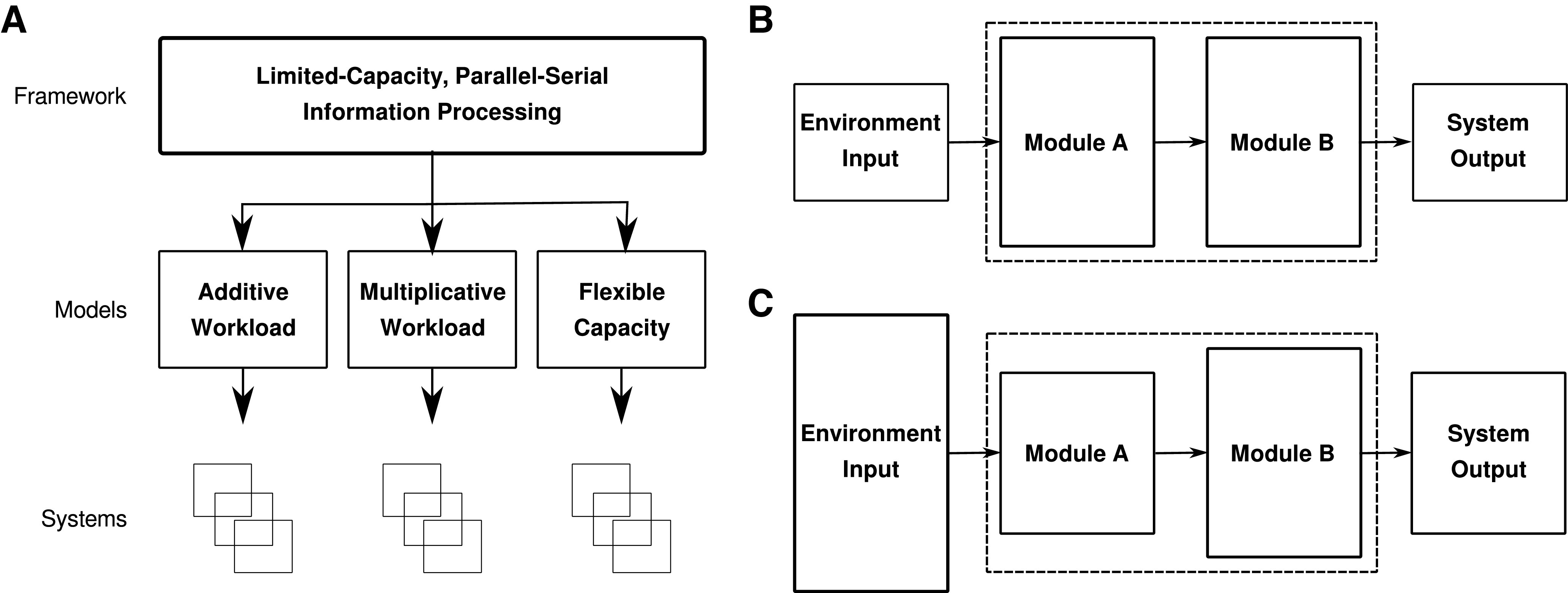
The limited-capacity, pipelined processing framework. ***A***, Our framework envisions information processing systems whose modules have limited processing capacities and that must process input serially while remaining capable of having different modules working on different inputs in parallel. Three separate models are considered within this framework, and we simulate a number of systems for each model to generate testable predictions. ***B***, For all limited-capacity, pipelined processing systems, the system output rate is equal to the environment input rate as long as the rate of environmental input is smaller than system capacity. In this case, the limitation on the output rate is outside the system. ***C***, Once the rate of environmental input exceeds system capacity, the limitation on the system’s output rate is within the system, and the system’s output rate is now equal to the capacity of the system’s most restricted module.

We developed three models within the limited-capacity, pipelined framework to explore the principles of information processing for interactive behavior. The time required for the perceptual, decision-making, and motor processes involved depends on the task’s complexity, but can be effectively reduced via parallel processing (Cisek and Kalaska, 2010; Thura and Cisek, 2014; Scott, 2016; Cisek and Thura, 2018; Cisek, 2019; Hommel et al., 2019). Our models each contain two modules that process input simultaneously but are connected in series, leading to pipelined processing. The ability for the modules to process input simultaneously matches the view that parallel processing for goal-directed actions is both more efficient and more biologically plausible (Pezzulo and Ognibene, 2012). While we commonly consider three processes for motor behaviors (perception, decision-making and motor control), the influence of pipelined processing can be easily observed with only two modules. The environment provides input to the system, each module processes input according to its own limited processing capacity, and the second module produces the system output (Fig. 3).

Kahneman posited that the amount of attention available increases with task difficulty. The brain performs processes in parallel by allocating attention between possible processes until demands exceed resources, at which point a degree of serial processing is forced (Kahneman, 1973). Since we are interested in peak performance, we focus on describing how peak performance occurs once task demands exceed the participant’s available attention. Our framework predicts that once the environmental input rate exceeds the system’s processing capacity, the system module with the smallest capacity will form a bottleneck (Fig. 3*B*). Building on the idea of limited-capacity, pipelined processing, we consider three separate models within this framework: Additive Workload, Multiplicative Workload, and Flexible Capacity. These models are distinguished by two characteristics: the effect of increased task demands and the nature of their processing capacities.

1. Task demands. Demands are either additive or multiplicative in nature. For systems from the Additive Workload model, more difficult tasks impose an additive workload on system modules: demands are placed on top of existing task input without modifying it.

By contrast, for systems from the Multiplicative Workload and Flexible Capacity models, more difficult tasks impose a multiplicative workload on system modules: demands increase existing task inputs by a common factor across the board.

1. Processing capacities. Systems from the Additive Workload and Multiplicative Workload models have fixed processing capacities for each module. They cannot adapt their capacity to changing task characteristics and require queueing when demands exceed capacity (Navon and Miller, 2002). This type of capacity represents neural resources that are fixed according to function.

Systems from the Flexible Capacity model have some of their overall capacity in the form of a shared pool of resources. These resources can be flexibly allocated between modules, meaning that sharing must occur when task demands exceed capacity (Navon and Miller, 2002). This is akin to neural resources being allocated to one specific process and improving its performance.

#### Task specifications

Our task environments were defined by the difficulty for each module to perform its processing task. We expressed this difficulty as a parameter *d* ∈ [0,1] where 0 indicated the highest level of difficulty and 1 indicated the lowest level of difficulty. A task environment was completely defined by the difficulty for the two modules’ processing tasks and can be thought of as a single point in the space [0, 1] × [0, 1].

We defined 100 task environments by varying the difficulty of each module’s processing from an initial value of 0.1 to a final value of 1.0, incrementing by a step of size 0.1. These task difficulty factors were deterministic and are given in Table 2. Since the task (1.0, 1.0) represents the easiest possible task environment, we use this as the baseline task for our comparisons.

**Table 2:** Grid of module difficulty factors

(0.1, 0.1)	(0.1, 0.2)	⋯	(0.1, 1.0)
(0.2, 0.1)	(0.2, 0.2)	⋯	(0.2, 1.0)
⋮		⋱	⋮
(1.0, 0.1)	(1.0, 0.2)	⋯	(1.0, 1.0)

Task environments were defined by the difficulty they presented to the two modules, with the first entry indicating the difficulty for Module A and the second entry indicating the difficulty for Module B.

Note that all task environments required both modules to perform their role in producing output. No task environment was made easier by dropping the requirement to process input via one of the modules.

#### System specifications

We generated individual systems from each of our three models. Module processing capacities were positive real numbers drawn from normal distributions with the means listed in Table 3 and a SD of 0.05 Hz. Each individual system was entirely defined by this pair of positive real numbers and can be expressed as a single point in the space ${\mathbb{R}}^{+}\times {\mathbb{R}}^{+}$.

**Table 3:** Grid of mean module capacities

(1.0, 1.0)	(1.0, 1.1)	⋯	(1.0, 3.5)
(1.1, 1.0)	(1.1, 1.1)	⋯	(1.1, 3.5)
⋮		⋱	⋮
(3.5, 1.0)	(3.5, 1.1)	⋯	(3.5, 3.5)

A system was randomly generated for each cell in the table where the first entry indicates the mean of the normal distribution that Module A’s capacity was drawn from and the second entry similarly indicates the mean of the normal distribution for Module B.

A system was generated for each cell in the table where the absolute difference in module capacities was less than a third of the first entry, preventing one module’s capacity from being completely dominated by the other’s bottleneck. This resulted in 324 systems. For systems from the Flexible Capacity model, each module’s processing capacity was reduced by 0.5 Hz to create a central pool of resources without increasing the system’s cumulative processing capability. This quantity was chosen arbitrarily to illustrate the principle of flexible allocation.

These initial module capacities were modified throughout the simulation according to that module’s task difficulty parameter. The final module capacities for systems in the Additive Workload model were calculated according to Equation 3, while final module capacities for both the Multiplicative Workload and Flexible Capacity models were calculated according to Equation 4:

(3) $${\text{Capacity}}_{\text{Final}}={\text{Capacity}}_{\text{Initial}}-1+d$$

e.g.,1.5=2-1 + 0.5

(4) $${\text{Capacity}}_{\text{Final}}={\text{Capacity}}_{\text{Initial}}\times d$$

e.g.,1=2×0.5

Module capacities for systems from the Flexible Capacity model were further modified by assigning the shared processing capacity to the modules. This shared capacity is assigned according to the following rules:

1. If the difference between module capacities is larger than the central capacity pool, assign all central processing capacity to the module with the smaller capacity.
2. Otherwise, assign the central processing capacity between the two modules so that the final module capacities are equal.

#### Simulation specifications

All 324 systems were simulated in all 100 task environments with 200 discrete frames recorded per second to match the recording fidelity of the Kinarm lab in the human experiments described below. Environmental input, capacity limits, and system output were all calculated every 100 frames and the input and output values for the intermediate frames were filled in using linear interpolation. The simulation calculated the environment’s steadily increasing input rate throughout the trial according to Equation 5, where *t* is the frame number, *n* is an additive noise factor drawn from the standard normal distribution N(0,1), and *input*(*t*) is the frame-specific input rate expressed in Hz. This ensured that every simulated trial started with an easy, data-limited phase and finished with an overwhelming, resource-limited phase:

(5) input(t)=0.5 + (0.025×t) + n/10.

To model fluctuating task demands over time, a frame-specific difficulty level was randomly generated for each module by sampling from a normal distribution with a mean of that module’s baseline difficulty and a SD of 0.05. Similarly, frame-specific module capacities were sampled from a normal distribution with a mean of the module’s base- line capacity and a SD of 0.05 Hz to model biological noise. These frame-specific difficulty levels and module capacities were then used to calculate the final module capacities for each frame according to Equation 3 or Equation 4 as appropriate.

The total output of a system for frame *t* was calculated as the minimum of the environment input, *input*(*t*), and the final module capacities, ${\text{Capacity}}_{\text{Final}}^{A}$ and ${\text{Capacity}}_{\text{Final}}^{B}$, with the restriction of being non-negative:

(6) $$output(t)=\text{max}(0,\text{min}(input(t),{\text{Capacity}}_{\text{Final}}^{A},{\text{Capacity}}_{\text{Final}}^{B})).$$

The per-frame input and output were recorded separately for each trial for further analysis.

### Code accessibility

The code/software described in the paper is freely available online at https://github.com/richard-moulton/Information-Bottlenecks-in-Rapid-Motor-Behaviour.

## Results

### Simulation predictions

**Figure 4.**
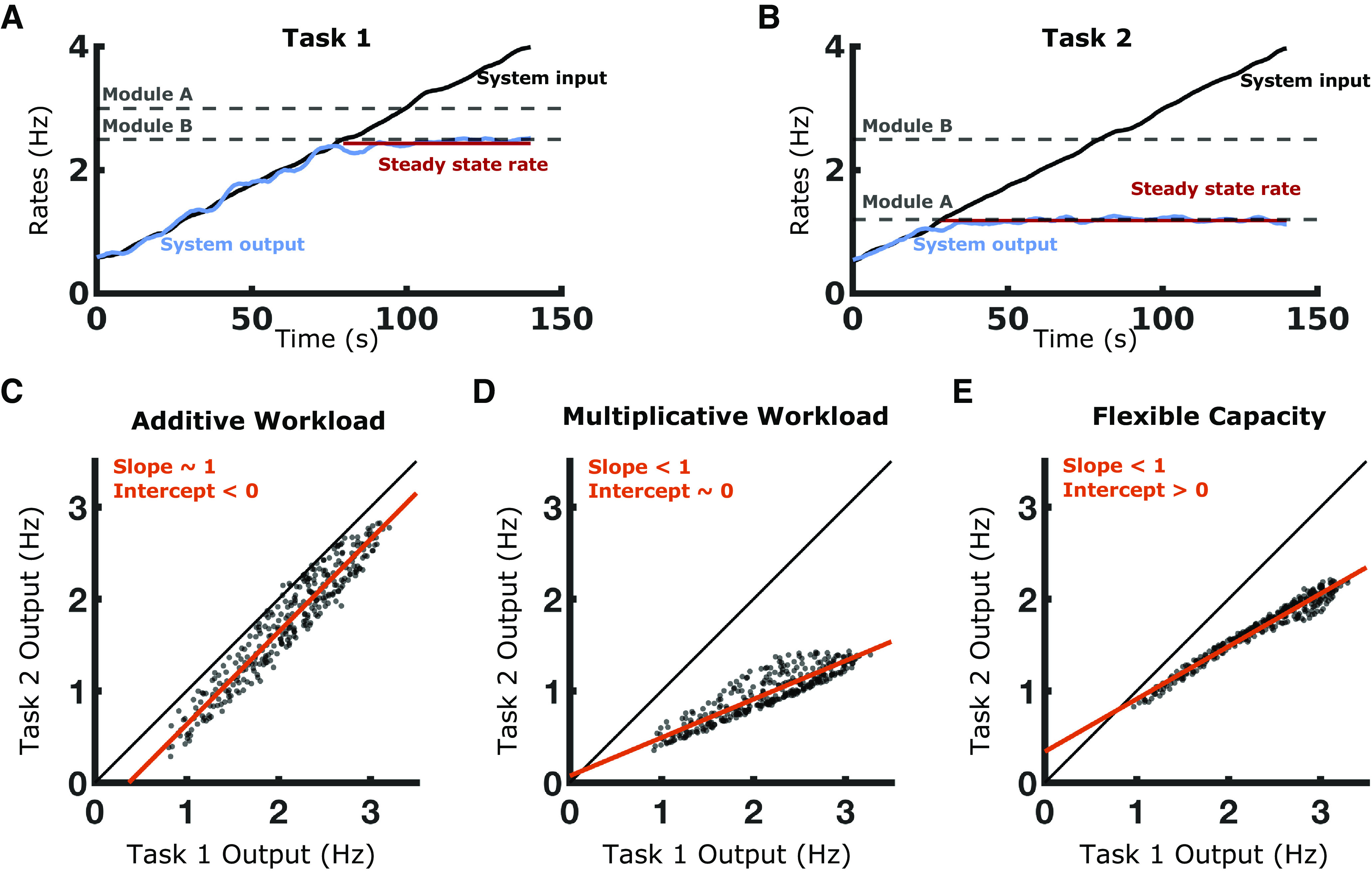
Predictions made by simulations. ***A***, Input rate (black) and output rate (blue) are shown over time for the baseline task. The processing capacity for each module is shown (gray) and the system’s steady-state output rate (red) is equal to the lower module capacity. ***B***, The same system performs a second task where Module A’s processing task is made more expensive; Module A’s capacity for this task is correspondingly reduced. ***C–E***, Scatter plot comparisons of systems’ steady-state rates are made for all three models of systems. Although 100 task environments were simulated, for clarity only a single comparison is presented between Task 1, the baseline task, and Task 2, a more difficult task. The unity line is shown in black and regression lines are shown in red.

A consistent observation for all three models was that systems reached a steady-state rate, at which point the same output was produced regardless of how much additional input the environment provided. This steady-state rate was the result of the system’s bottleneck: the smaller module capacity (in this example, Module B) aligned with the steady-state rate and solely determined the system’s overall output (Fig. 4*A*). A task with different difficulties, however, could see another module (Module A) become the system’s bottleneck (Fig. 4*B*).

Examining the different types of task demands, we observed that an additive workload leads to all systems being affected to the same degree. This results in regression slopes near 1, which indicates that less capable systems would be unable to perform tasks that were too difficult (Fig. 4*C*). By contrast, a multiplicative workload leads to the best-performing systems being most affected by increased task demands. This results in regression slopes <1, but all systems are able to perform the more difficult task (Fig. 4*D*,*E*).

Assessing the different models, we observed that structural bottlenecks lead to systems being affected by increased difficulty up until 0 Hz. This results in y-intercepts near or below 0 Hz (Fig. 4*C*,*D*). The existence of a shared pool of resources allows the worst-performing systems to mitigate task demands, which increases the average performance across all systems. For example, systems in the Flexible Capacity model that had a high processing rate for Module A largely used their central resources in Task 1 to improve their Module B capacity and maximize performance. When Task 2 reduced Module A’s performance, these systems were able to redistribute between modules, mitigating these new demands. By contrast, systems with a high processing rate for Module B had largely used their central resources to improve Module A’s capacity in Task 1 and additional resources were therefore not available during Task 2 to counter the reduced performance of Module A. The overall effect of central resources was a positive y-intercept and the linear regression crossing the unity line in Quadrant I of the Cartesian plane (Fig. 4*E*). Note that the predictions made by the Additive Workload and Flexible Capacity models are only valid to the right of the unity line in Quadrant I: neither model predicts output that is either negative or increased for the more difficult task.

All three models predict that systems will produce lower steady-state rates for more difficult tasks, but they make diverging predictions about how this occurs:

1. The Multiplicative Workload and Flexible Capacity models predict increasing task difficulty will affect the best-performing participants more than worse-performing participants, the slope for the linear regression will be less than 1.

By contrast, the Additive Workload model predicts that all participants are equally affected by increased task difficulty, the slope for the linear regression is equal to 1.

1. The Flexible Capacity model predicts that there is a positive level of performance at which participants will be unaffected by this additional task difficulty, the y-intercept for the linear regression is positive.

The Additive Workload and Multiplicative Workload models predict that equal performance between tasks will only occur at 0 Hz. This demonstrates an inherent advantage to developing processing capacity in the form of shared resources because it reduces the effect of variable task difficulties on individual system performance.

Only the Flexible Capacity model predicts that participants’ drop in performance with increased task demands will fall more heavily on more skilled participants and that some participants will actually be unaffected by increased task demands. We will show over the following sections that only the Flexible Capacity model is consistent with human performance in our rapid motor behavior tasks.

### Experiment 1: Object-Hit and Object-Hit-and-Avoid

For OH and OHA, our two-module system roughly represents the perceptual and decision processes with Module A and the motor processes with Module B. Although the baseline task OH does place demands on all processes, the bottleneck is likely the motor system’s ability to hit targets. Compared with OH, OHA adds additional pressures to identify, select and hit specific targets and avoid distractors. This shifts the bottleneck toward the perception/decision module, since objects’ shapes must be reliably perceived and an arbitrary decision rule applied to assess target/distractor status.

We established a ROPE for differences in steady-state rates of (–0.30, 0.30 Hz) to account for the between-trial variability for participants performing OH (see Materials and Methods, Statistical analysis). Steady-state rate differences between OH and OHA that fall within this region are considered to be practically equivalent to 0 Hz. This intertrial variability is independent of performance level, allowing us to use the same ROPE for all participants.

#### Performance decreases with task difficulty

**Figure 5.**
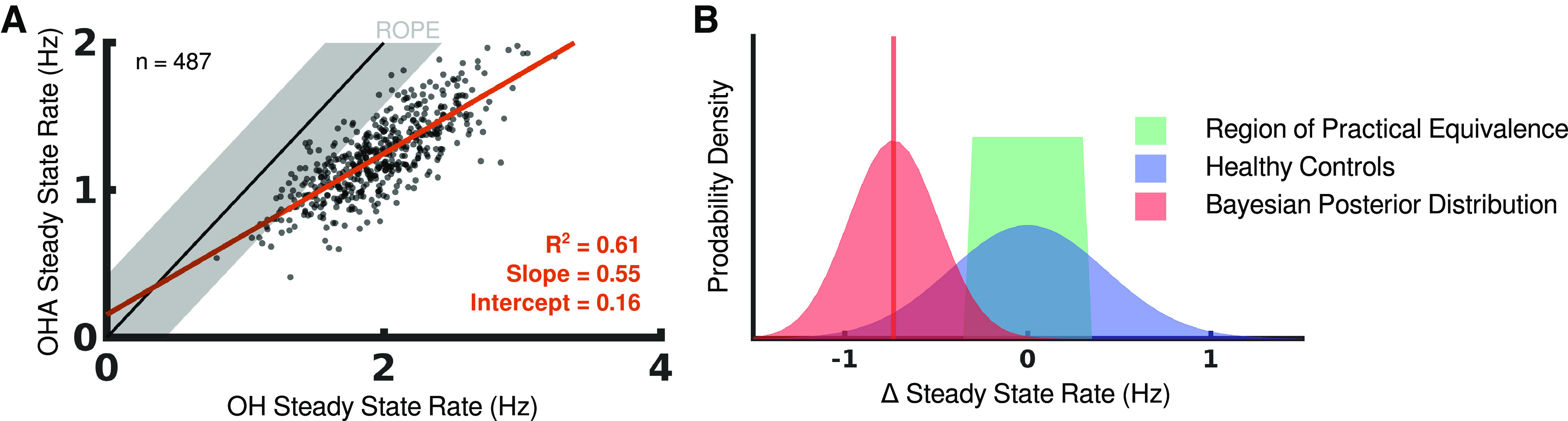
Comparing performance between OH and OHA. ***A***, Participants are scatter-plotted according to their steady-state rates in OH and OHA. The unity line is shown in black and is extended by the region of practical equivalence in gray. Regression for participants’ performance is shown in red. ***B***, Probability distribution functions are plotted to represent the region of practical equivalence for OH (green), the distribution of steady-state rates seen in healthy control participants for OH (blue), and the posterior distribution for differences in steady-state rates between OH and OHA (red). The mean of the posterior distribution is highlighted by the red vertical line.

Scatter-plotting participants’ steady-state rates in OH and OHA highlights that the latter is the more difficult task (Fig. 5*A*). The mean difference in steady-state rates between the tasks is 0.733 Hz, which is 2.4 times further from 0 Hz than the boundary of the ROPE (Fig. 5*B*). Taken over the course of a whole trial, ∼140 s, this represents nearly 103 fewer targets hit by a participant. Although OHA does have fewer targets than OH, this cannot account for the entire drop in performance.

To determine whether participants’ steady-state rate differences are significantly different from 0 Hz, we modelled these differences as being drawn from a normal distribution with mean *μ* and SD *σ*, then used a Markov chain Monte Carlo (MCMC) method to estimate these parameters (Fig. 5*B*; for details, see Materials and Methods, Statistical analysis). The 95% HDI for *μ* is entirely below the ROPE for the OH task, indicating that the mean difference in participants’ steady-state rates between OH and OHA is practically different from 0 Hz. Notably, most participants perform OH before OHA so any population-level intertask learning effect would be in favor of performance in OHA.

#### The size of performance drop scales with ability

**Figure 6.**
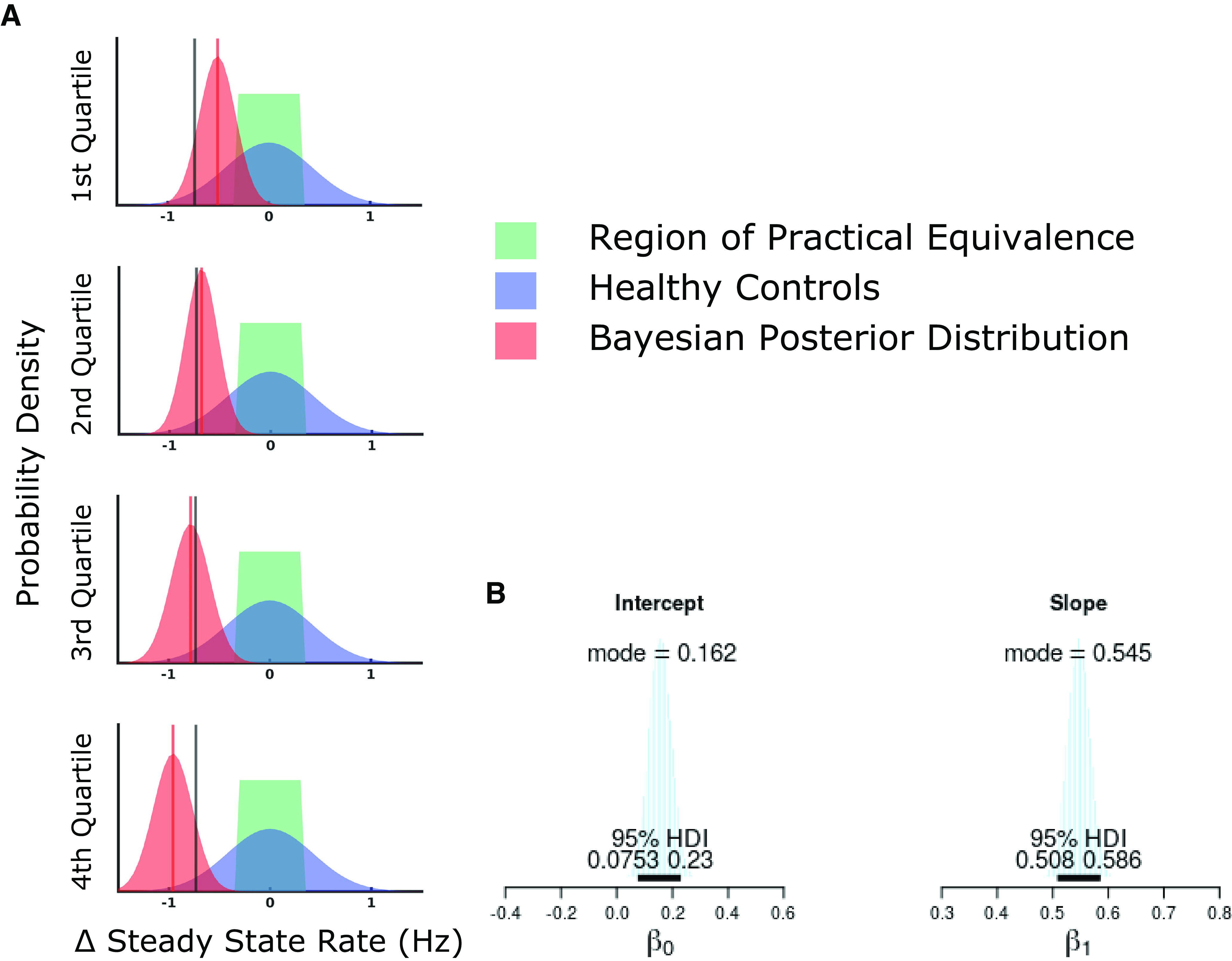
Drops in performance between OH and OHA are especially significant for better performers. ***A***, Participants are grouped by their performance quartile in OH and the analysis from Figure 5*B* is repeated. The mean of the posterior distribution is highlighted by the red vertical line for each quartile and is compared with the mean of the posterior distribution for all participants, shown with the black vertical line. ***B***, Distributions of value estimates are shown for the intercept and slope parameters from the Bayesian linear regression relating participants’ steady-state rates for OH and OHA. Specifically highlighted are the mode value of the distribution and the distribution’s 95% high-density interval.

We split participants into quartiles based on their steady-state rate in the OH task and performed the same Bayesian data analysis for each quartile. We found participants in the lower quartiles are least likely to show a significant drop in performance while participants in the top-performing quartile are most likely to show a large drop in performance (Fig. 6*A*); in fact, the mean difference in steady-state rates for participants in the top-performing quartile is outside the 95% confidence interval for healthy control participants in the OH task.

We used Bayesian linear regression to test our models’ predictions (see Materials and Methods, Statistical analysis). We estimated the *t*-distribution’s scale and normality parameters directly to confirm that a linear model reasonably linked participants’ steady-state rates. The *t*-distribution’s centrality parameter was estimated as a linear function of the participant’s steady-state rate in the OH task according to Equation 7:

(7) $$SSR_{OHA}∼t_{\nu }(\mu =\beta_{0}+\beta_{1}SSR_{OH},\sigma ).$$

The estimates for the linear regression parameters are shown in Figure 6*B*.

We estimated the value for the linear regression slope parameter, *β*_1_, as 0.545 (95% HDI 0.508–0.586). This is clearly far from 1: not only is the 95% HDI for this estimate entirely below the ROPE, (0.95, 1.05), but so is the entire distribution of estimates. In the context of the tasks, a regression slope of 0.545 indicates that if a participant improved their performance in OH by 10 extra targets, we would only expect them to improve in OHA by five or six extra targets. This estimate range for *β*_1_ provides convincing evidence that skilled participants are more affected by an increase in task difficulty than less skilled participants. This is consistent with the Multiplicative Workload and Flexible Capacity models, but contradicts the Additive Workload model.

#### Some participants are unaffected by additional task difficulty

The observation that participants who perform best on OH experience the largest drop in performance for OHA is especially striking because we see that the worst participants on baseline tasks often perform within the ROPE when shifting to OHA—that is many of the worst participants perform just as well on the harder task as they would be expected to perform on a second trial of the baseline OH task (Figs. 5*A*, 6*A*). To test this observation quantitatively, we revisit our Bayesian linear regression’s intercept parameter, *β*_0_, and determine whether it is larger than 0 Hz; our ROPE around 0 Hz was (–0.12 Hz, 0.12 Hz). The estimate for *β*_0_ is centered on 0.162 Hz (95% HDI 0.075–0.230 Hz) with 81.4% of the estimate’s probability mass greater than the ROPE. This provides strong evidence that there is a performance level at which participants are not affected by an increase in difficulty in the task, some level of ability exists independent of task difficulty. This result is consistent with the Flexible Capacity model, but contradicts the Additive Workload and Multiplicative Workload models.

### Experiment 2: Turbo Object-Hit and Turbo Object-Hit-and-Avoid

**Figure 7.**
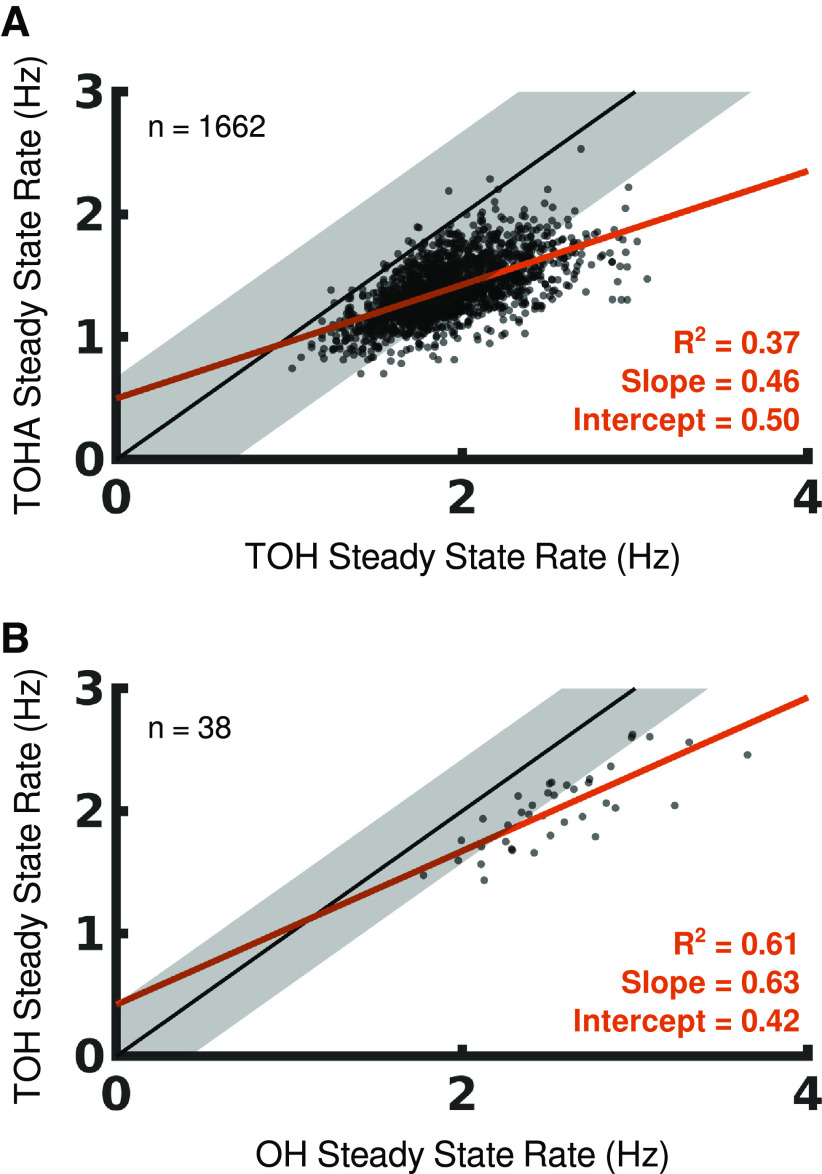
Comparing performance between tasks. Participants are scatter-plotted according to their steady-state rates in three cross-task comparisons: (***A***) TOH and TOHA and (***B***) OH and TOH. The unity line for each comparison is shown in black and extended by the region of practical equivalence in gray. Regression for participants’ performance is shown in red.

In this experiment, we considered participants who performed the original tasks’ “turbo” variants (Mang et al., 2018). We saw a systematic shift downwards in steady-state rates from TOH to TOHA (Fig. 7; Table 4).

**Table 4:** Experimental results

	Experiment 2	Experiment 3
	TOH-TOHA	OH-TOH
Δ SSR (Hz)	**0.532 (0.520–0.545)**	**0.530 (0.450–0.614)**
*β*_1_ (1)	**0.464 (0.436–0.495)**	**0.623 (0.461–0.809)**
*β*_0_ (Hz)	**0.494 (0.436–0.550)**	0.418 (−0.036–0.853)

The estimated difference in steady-state rates and Bayesian linear regression parameter estimates (*β*_1_ and *β*_0_) are given for Experiments 2 and 3. The 95% high-density interval for each estimate is given in parentheses. Estimates are bolded to indicate that their 95% HDI is completely outside of the associated ROPE.

A similar quartile-based effect is seen for this pair of tasks (Fig. 7*A*). Quantitatively, the 95% HDI for the slope parameter is entirely less than the ROPE around 1 and the 95% HDI for the y-intercept parameter is entirely greater than the ROPE around 0 Hz (Table 4). These results are in line with Experiment 1 and are consistent with the Flexible Capacity model.

### Experiment 3: Object-Hit and Turbo Object-Hit

Experiments 1 and 2 both assessed pairs of tasks where the additional difficulty was a result of the distractor objects, thus the dimension of increased task difficulty was the same for both experiments. In Experiment 3, we assessed our models against the performance of participants who performed both OH and TOH. In this pair of tasks, the increased difficulty occurs because the participant must use substantially smaller paddles to hit faster moving targets. This allows us to investigate whether the evidence found for limited capacity, pipeline processing generalizes beyond cases where an arbitrary decision rule is added.

We observed that steady-state rates were consistently lower in TOH than in OH (Fig. 7; Table 4). Comparing SSRs between these two tasks produces the same quartile-based effect (Fig. 7*B*) and the 95% HDI for the slope parameter is entirely less than the ROPE around 1, consistent with multiplicative task difficulty. Although the 95% HDI for the y-intercept parameter overlaps with the value of 0 Hz, >90% of the estimate’s probability mass is greater than the ROPE (Table 4). This lends moderate support for the Flexible Capacity model.

## Discussion

We show that human performance in ongoing, rapid motor behaviors is consistent with a limited-capacity, pipelined processing framework. We found that task demands increased multiplicatively, with the greatest impact on those with better baseline performance. Our results further suggest that neural resources are flexibly allocated between processes to maximize performance.

### Principles of pipelined processing

Our systems featured two modules connected in serial, but their ability to process input in parallel is a massive advantage over strictly serial processing: output increases by a factor of *n* for an *n*-module system with similar capacity modules. Our framework demonstrates that once processing limits are reached, task and system characteristics conspire to produce bottlenecks which result in the steady-state behavior that is the marker of an overwhelmed system. We distinguish our systems’ pipelined processing from parallel-distributed processing because of the serial nature of these processes: perception must precede deliberation, which in turn must precede deliberate action, even if these processes do overlap and interact. These serial aspects open the door for any one aspect to become the limiting factor on the system’s output rate by becoming a bottleneck.

We investigated additive and multiplicative task demands, e.g., adding a framing phase versus increasing each target’s difficulty, with our models showing that multiplicative demands affect the best performing systems the most in absolute terms. This suggests that performance drops say more about a task’s demands than the system performing it. Concretely, if one participant suffers a larger performance drop than another, we should not necessarily conclude that the former participant found the new task harder than the latter participant. Instead, the former participant might have had a larger portion of their performance that was vulnerable to redistributing the shared resources in response to changed task demands.

We also contrasted structural bottlenecks against shared resources. A system with structural bottlenecks is at the mercy of task demands and will lose ground to increased demands until 0 Hz. The ability to shift resources between processes allows the system to safeguard performance against increased task demands instead.

### Human behavior matches that of our model

Human performance across behavioral tasks matched the Flexible Capacity model’s characteristics in a number of ways.

First, we saw evidence of parallel processing as in parallel models for goal-directed actions (Pezzulo and Ognibene, 2012). Tasks such as OH reward parallel processing because of repeated stimuli and sequential decision-making. Consider the following reaction-time chain: 150 ms to make a go/no-go decision using an arbitrary rule based on perceptual stimuli (Thorpe et al., 1996), 600 ms to accumulate evidence between two alternatives (Ratcliff and McKoon, 2008), and 1000 ms to make a simple 35 cm arm movement (Morasso, 1981). If these processes occurred serially, then the sum of 1.75 s per object would limit steady state rates in OHA to ∼0.57 Hz. Most participants comfortably exceed this ceiling (Fig. 5) with the top performers approaching a steady-state rate of 2 Hz. This improved performance represents a clear evolutionary incentive for different brain functions to process in parallel, as has been proposed for the human sensorimotor system (Cisek and Kalaska, 2010; Cisek, 2019). In addition to the benefit of speed, parallel processing also allows information from current actions to shape an ongoing decision-making process (Lepora and Pezzulo, 2015).

Second, we saw that additional demands in behavioral tasks have a multiplicative nature, leading to the best participants seeing the largest drop in performance on harder tasks. Although we might expect that the most skilled participants could minimize the effect of additional demands, our results demonstrate that the most skilled participants are most affected precisely because they are processing the most targets to begin with. The worst performers are completely unaffected by the increased difficulty of targets they were not hitting anyways.

Third, we saw that the least skilled participants were often able to reproduce their steady-state rates in more difficult tasks and showed no practical drop in performance. This did not come from underlying skill but is consistent with allocating shared resources between processes according to task demands. We do not believe that this effect can be explained by participants regressing to the mean, since even if the outlier performers did regress, we would still expect the whole sample to include relatively extreme performance levels on the more difficult task. This suggests that there is a base level of motor performance that is very resilient to outside task demands.

### Comparing OH tasks with other tasks

Perceptual, decision-making, and motor control processes have historically been studied using reaction-time tasks, including inter-response interval tasks such as key presses after sequential stimuli (Kahneman, 1973). In the motor decision literature, “decide-then-act” tasks such as motor lotteries (Trommershäuser et al., 2008; Wu et al., 2009; Jarvstad et al., 2013; Neyedli and Welsh, 2013, 2015; Neyedli and LeBlanc, 2017), foraging tasks (Diamond et al., 2017), and intercept tasks (Barany et al., 2020; Fooken and Spering, 2020) have been used to characterize decisions about movements. These tasks have used external time limits to provoke reaction-time like processing to measure the brain’s capacity in motor decisions, but provide minimal opportunity for the brain to exploit its ability for parallel processing.

Rapid motor behavior tasks allow the same motor decision capabilities to be explored, but in the context of ongoing, interactive behavior that results in interference between processes. A participant’s steady-state rate acts as an ongoing and sustained reaction time, which is useful for “decide-while-acting” tasks where humans have been shown to prioritize the global reward rate (Bogacz et al., 2010; Balci et al., 2011). As a practical matter, rapid motor behavior tasks also include a speed-up benefit over other experimental tasks. As a comparison, 300 separate objects can be presented to the participant and either acted on or not within 3 min; by contrast, 300 presentations in a motor lottery task, allowing for 5 s of reset time in between trials, would require 30 min.

### Implications for attention

A key feature of our Flexible Capacity model is the central pool of resources that can be flexibly allocated to whichever module is most stressed by the task’s demands. In the brain, this pool of resources is commonly termed *attention*. Although notoriously difficult to define, one description of attention from the literature is that it is the brain’s ability to selectively process multi-channel sensorimotor input when continuous, interactive behavior is required to achieve our goals (Hommel et al., 2019). This ability is goal-related, e.g., people will not notice the unexpected gorilla in a scene when instructed to count the number of basketball passes they observe (Simons and Chabris, 1999). Importantly, the degree to which an individual can pay attention acts as a capacity limit on their ability to act. Not all neural processes require attention, Schneider and Shiffrin distinguish between automatic and controlled processes (Schneider and Shiffrin, 1977; Shiffrin and Schneider, 1977) and Kahneman distinguishes between System 1 and System 2 (Kahneman, 2011). But processes that do require attention are subject to capacity limits and are prevented from occurring freely in parallel. These are the processes which are subject to constraints in our model.

Kahneman proposed that attention is generated through mental effort and shared across tasks to meet demands and allow parallel processing. Once the maximum attention capacity is generated, the brain’s neural processes interfere with each other’s demand for resources. This leads to some goals being delayed or dropped to permit others to be achieved (Kahneman, 1973). Our results support the existence of a shared pool of neural resources, though we do not speculate about how this pool is made available to neural processes. In all three experiments there is significant support for our linear regression’s y-intercept term being larger than 0. This is predicted by the Flexible Capacity model and indicative of shared resources being re-deployed according to changed task demands. By contrast, the Multiplicative Workload model cannot explain this since structural bottlenecks cannot, by definition, adapt. Our results extend Kahneman’s conception of neural processes competing with one another for resources beyond the “decide-then-act” paradigm by providing evidence for the same type of interference effects during “decide-while-acting” tasks.

We propose that one of the functions of attention during motor tasks is to compensate for goal-related demands. Since attention is a finite resource, this enforces a selectivity between processes that directly explains the serial processing effects that occur in limited capacity systems (Kahneman, 1973). This selectivity in assigning attention between processes causes information bottlenecks to occur between perception and action. The types of rapid motor behavior subtasks requiring attention include: actively searching for decision-relevant information (Orquin and Mueller Loose, 2013), storing perceived stimuli into visual short-term memory (Künstler et al., 2018), planning movement toward different targets (Gallivan et al., 2011), and deciding between alternative motor actions (Haith et al., 2015), especially under uncertainty (Brandstätter and Körner, 2014) or when precise selection is required (Franconeri et al., 2007). Associating behavioral bottlenecks with attention demands is consistent with a number of stimulus response frameworks including: the selection of inputs and selection of outputs (Treisman, 1969), the stimulus set and response set (Broadbent, 1971), and figural emphasis and response selection (Kahneman, 1973).

Considering that these bottlenecks occur when processes must compete for limited attention, it is important to note that controlled processes can be learned to the point where they become automatic processes and thereby reduce their demand for resources. Such learning reduces the instantaneous demands on shared resources during tasks and allows this limited capacity to be directed toward processing important stimuli and controlling increasingly complex responses (Shiffrin and Schneider, 1977). This kind of learning and the dedication of controlled and automatic processes to specific kinds of tasks may also support hierarchical control for motor actions. For example, there is evidence that skilled typists can type roughly twice as fast with two hands as they can with a single finger, but for student typists this is reversed (Gentner, 1983). A possible explanation for this type of effect is that each hand is controlled by separate hierarchical control processes which alternate between the controlled process of sequence planning and the automatic process of sequence execution (Sternberg et al., 1990). Our model is consistent with this explanation, since we can model a shift toward automatic processes as an increase in that module’s capacity. Although we have not previously found evidence for the strategic alternation of hand movements in the OH family of tasks (Moulton et al., 2022) there is clear scope for our model to be extended to account for the bimanual nature of many rapid motor behaviors tasks.

In interpreting the results of Experiment 3, we might expect that additional stresses on a bottleneck process could not be remedied by reallocating shared resources since they are already devoted to the bottleneck process during the easier task. Indeed, the y-intercept term *β*_0_ for Experiment 3’s linear regression is the only parameter whose 95% HDI overlapped the associated ROPE. The estimated y-intercept of 0.418 Hz does suggest some effect, however, and this would be possible if the initial distribution of shared resources was split between processes, leaving some available to be reassigned once the bottleneck process is further stressed. Related to this possibility is the question “what proportion of the brain’s processing capacity is a result of the brain’s different regions versus as a result of a central pool of neural resources?” This is an important parameter in shared resource models (Navon and Miller, 2002) and could be the subject of future research. Another question posed by shared resource models is the degree to which resources can be shared across all processes. Theories that posit the existence of multiple resources propose that different pools of resources are available to processes based on the processing stage, encoding, and modality of the process (Wickens, 2008). Task difficulty in these theories reflects both the degree to which individual processes exhaust their own resources and the amount of competition that exists for common resources (Navon and Gopher, 1978). Future work could aim to characterize the source and degree of difficulty in rapid motor behavior tasks using performance operating characteristic curves (Navon and Gopher, 1978, 1979; Wickens, 1981).

### Future applications

Our Flexible Capacity model assumed that all systems have a fixed number of shared neural resources, regardless of individual module capacities. Task comparisons could establish the proportion between structural and shared neural resources for rapid motor behavior tasks as well as the interparticipant variability for this proportion. Our model also assumed that shared neural resources can be split across processes arbitrarily and dynamically in response to changing task demands. The validity of this assumption could be tested by using tasks that measure the effects of split attention and shifts of attention on participants’ steady-state rates. Our model could also be extended to incorporate well-known cognitive systems, such as working memory, with a view to examining how these specific systems interfere with other neural processes.

Our model may also be useful for interpreting results in clinical settings. Specifically, stroke may impact motor function and/or cognitive function so that changes in performance between OH and OHA may be much more variable: slow performance in OH could lead to no change in OHA (motor impairments only), or dramatic impairment in OHA (motor and cognitive/perceptual impairments). The general prediction is that any neurologic disease or injury where attention helps the individual counter an impairment will leave that individual susceptible to performance-impacting bottlenecks if behavioral demands are increased, such as a person with difficulties walking but now having to also navigate in a crowded room.

Our model also suggests that athletic coaches and rehabilitation therapists should determine an individual’s bottleneck process to allow targeted training that will produce outsized rewards on improved performance. This is the pay-off of learning motor skills, e.g., the autonomous stage of motor learning (Schmidt and Wrisberg, 2004). Since motor skills involve perception, decision-making, and motor control processes (Wolpert et al., 2011), reducing a particular process’s requirement for attention frees up shared resources to assist another process. This is the initial challenge of learning to drive where attentional demands must be used to perform the basic requisite motor actions (i.e., switch on the turn indicator, press the brake and turn right) at the expense of attending to perceptual and decision processes. This is analogous to the benefit seen in cognitive decision-making when shifting from deliberative to procedural decision-making (Redish, 2013). Similarly, this principle is exemplified by the fact that airplane control instruments, such as joysticks and pedals, are easy to use, ensuring the pilot can focus on perceiving instruments, listening to air-traffic control, and/or directing the co-pilot.

## Synthesis

Reviewing Editor: Christoph Michel, Universite de Geneve Decisions are customarily a result of the Reviewing Editor and the peer reviewers coming together and discussing their recommendations until a consensus is reached. When revisions are invited, a fact-based synthesis statement explaining their decision and outlining what is needed to prepare a revision will be listed below. The following reviewer(s) agreed to reveal their identity: NONE.

Both reviewers consider the paper interesting and well done. Their comments are mainly conceptual and concern some of the interpretations. The first reviewer suggest to better highlight the novelty of the present work with respect to existing theories. The second reviewer would like to see further discussions concerning the influence of motivation on task performance. The detailed reviewer’s comments are given below. A point-by-point response to each of the points raised is requested.

Reviewer 1:

This manuscript offered a revisit of older theoretical accounts for the control of rapid motor behaviors. Using modeling and some existing data from standard tasks using the KINARM, the authors argue for a limited capacity, parallel-serial processing model where resource allocation can occur flexibly.

The authors suggest that the methodological approach (i.e., dynamic tasks with KINARM vs. traditional RT paradigms) is more appropriate to address ongoing control of fast-action motor behaviors.

The paper is well written and interesting to read. Frankly, it reminded me of issues the field spent considerable time contemplating quite some time ago in the context of psychological refractory period, visual and memory search paradigms as well as the concomitant theoretical descriptions of attention advanced by Sternberg, Shiffrin & Schneider, Kahnemann and many others.

I mention this because one of the shortcomings of this work in my opinion is that the novelty or unique contribution of this work is not well highlighted in the manuscript. In a way, it is a “revisit” or reminder of the importance of “attention” or “resource allocation” in contexts in which the human operator faces increasing performance demand. The outcome/contribution from this work is an account that combines the two primary approaches that were considered for performance breakdown in the earlier literature. Specifically, structural limitations (i.e., bottlenecks, see psychological refractory period) or capacity limits (see structural vs. central interference issues). Going forward, I think the authors need to more explicitly addresses how this work advances traditional accounts and offers something unique.

In doing so, the following are a few issues the authors might address or at least consider:

(a) is there a critical distinction between parallel and parallel-serial processing? For example, in your version in which two modules are modelled you suggest one might envision this as perceptual and decision processes occurring in one module while motor processes are resolved in a separate module. With this in mind, would resolving perceptual and decision uncertainly but not motor result in same/different outcomes as solving perceptual and motor uncertainty but not decision demands?

(b) is serial-parallel processing different from parallel-distributed processing the latter of which might be more common nomenclature when this work is extended to consider neural implementation, an idea floated in parts of the paper.

(c) I’m not convinced that the tasks used within the context of the KINARM are easy to distinguish based on the specific processing demands. While the “avoid” protocol likely loads on a decision process I am not so sure what the turbo version does. Again, I’m reminded of how carefully PIs selected experimental manipulations (i.e. conditions) to target specific processes to be clear what increased demand (see also additive factors methods). I’m not sure this is clear with these tasks, maybe it’s not that important right now. However, will it become important as this work is advanced?

(d) Is a single shared and flexible use resource the only capacity model to consider? I seem to recall that there was some thought that resources might be process specific. For example, I believe Wickens and others forwarded a multiple resource account some time ago.

Reviewer 2:

This paper evaluates how cognitive (i.e., attentional resources) are allocated across tasks (easy vs hard), comparing two different types of performers (high vs low). They found that behavior respects a Flexible Capacity model, in which sources can be allocated flexibly across different (motor, perceptual) modules depending on the demands of the task.

This paper was nicely contextualized, the results were clear, and the conclusions were appropriate; I only have two minor suggestions for the authors to consider discussing.

1) The critical result that differentiated the multiplicative vs flexible capacity model was that the (inferred) intercept of the scatter plot was none-0 (Figure 5A). However, this seems to make a radical prediction that there would be, in theory, individuals who exert greater output in task 2 (the harder task) compared to task 1, if we follow the line close to (0, 0). Why were there no individuals here? Are behavioral manipulations that can reveal such behavior?

2a) The authors argue that this is not motivation, but I don’t quite follow the reasoning on page 10 “if motivation played a significant role, then we would expect to see some participants exhibit substantial increases or decreases in hitting rate during their overwhelmed phase?” Have the authors shown that there are no substantial fluctuations in the overwhelmed phase?

2b) Is it possible that the steady-state reflects the level of motivation participants set for both easy and hard tasks? That is, if you incentivized participants with more money, would the participants remain at the same steady state (more persistent trait-like quality), or could financial incentives motivate participants to modulate their steady-state rate (incidental state-like quality)? If the latter, then how do the authors conceptualize the possibility of this steady-state reflecting a self-selected ‘motivational’ threshold rather than a true sensorimotor/attentional ‘information bottleneck’?

Altogether, I thoroughly enjoyed reading the paper, and am envious of their sample size!

## Author Response

Dear Dr. Michel,

We have received your letter regarding our manuscript “ Capacity limits lead to information bottlenecks in ongoing rapid motor behaviours “ (Submission eN-NWR-0289-22). We thank the reviewers for their thoughtful and constructive comments.

Given your decision that this manuscript is potentially suitable for publication in eNeuro, we have revised our manuscript to address the reviewers’ feedback. Most notably we have:

1. Incorporated references and comparisons to additional works from the literature addressing limits on human performance; and

2. Revised paragraphs where the reviewers were unclear on our meaning.

Additionally, a thesis committee member for the first author suggested that the Methods sections related to our simulations be reorganized to clarify our specifications. This reorganization involved sections 2.5.1, 2.5.2, and 2.5.3 in the manuscript and does not materially alter these sections’ content.

We believe that this revision improves the manuscript but could revert to our originally submitted

Methods section if that is the editor’s preference.

Our response to the editor’s and reviewers’ comments follows, with particular emphasis on how we have revised our manuscript. Original comments appear in bold and our responses appear inline; line numbers refer to the latest revision.

We hope that you find our resubmission suitable for publication and look forward to hearing from you soon.

1. Synthesis Statement for Author

Both reviewers consider the paper interesting and well done. Their comments are mainly conceptual and concern some of the interpretations.

We are grateful to the reviewers for their kind comments.

The first reviewer suggest to better highlight the novelty of the present work with respect to existing theories.

As described below in our comments to each reviewer, we now refer to additional works addressing human performance limits. In particular, we now highlight the novelty of the present work with respect to Kahneman’s model of attention as mental effort, which greatly influenced the development of our own thinking.

Our results extend Kahneman’s conception of neural processes competing with one another for resources beyond the ``decide-then-act” paradigm by providing evidence for the same type of interference effects during ``decide-while-acting” tasks. -- lines 637-640

The second reviewer would like to see further discussions concerning the influence of motivation on task performance.

As described below, we have answered the reviewer’s questions regarding the influence of motivation on task performance and have revised the relevant paragraph in our paper for clarity.

The detailed reviewer’s comments are given below. A point-by-point response to each of the points raised is requested.

Our response to the reviewers’ comments follows, focusing on the revisions we have made to our manuscript. Original comments appear in bold, and our responses appear inline.

2. Reviewer 1

This manuscript offered a revisit of older theoretical accounts for the control of rapid motor behaviors. Using modeling and some existing data from standard tasks using the KINARM, the authors argue for a limited capacity, parallel-serial processing model where resource allocation can occur flexibly.

The authors suggest that the methodological approach (i.e., dynamic tasks with KINARM vs. traditional RT paradigms) is more appropriate to address ongoing control of fast-action motor behaviors.

Thank you for this high-level summary, we are excited by the possibility for this methodology to be useful in further exploring the control of rapid motor behaviours.

The paper is well written and interesting to read. Frankly, it reminded me of issues the field spent considerable time contemplating quite some time ago in the context of psychological refractory period, visual and memory search paradigms as well as the concomitant theoretical descriptions of attention advanced by Sternberg, Shiffrin & Schneider, Kahnemann and many others. I mention this because one of the shortcomings of this work in my opinion is that the novelty or unique contribution of this work is not well highlighted in the manuscript. In a way, it is a “revisit” or reminder of the importance of “attention” or “resource allocation” in contexts in which the human operator faces increasing performance demand. The outcome/contribution from this work is an account that combines the two primary approaches that were considered for performance breakdown in the earlier literature. Specifically, structural limitations (i.e., bottlenecks, see psychological refractory period) or capacity limits (see structural vs. central interference issues). Going forward, I think the authors need to more explicitly addresses how this work advances traditional accounts and offers something unique.

We agree that there is a rich literature addressing the topic of limits on human performance, whether due to attention, general neural resources, or structural constraints and thank you for the list of relevant researchers.

With regards to Kahneman’s model of attention as effort, we do highlight that we are most interested in participants’ performance once they exert maximum effort:

The brain performs processes in parallel by allocating attention between possible processes until demands exceed resources, at which point a degree of serial processing is forced [31].

Since we are interested in peak performance, we focus on describing how peak performance occurs once task demands exceed the participant’s available attention. -- lines 270-274

We have revised our discussion to highlight that Kahneman’s model was developed on the basis of experiments in the “decide-then-act” paradigm of reaction-time tasks, while our work extends these ideas to assessing performance on tasks in the “decide-while-acting” paradigm of ongoing motor actions:

Kahneman proposed that attention is generated through mental effort and shared across tasks to meet demands and allow parallel processing. Once the maximum attention capacity is generated, the brain’s neural processes interfere with each other’s demand for resources. This leads to some goals being delayed or dropped in order to permit others to be achieved [31]. Our results support the existence of a shared pool of neural resources, though we do not speculate about how this pool is made available to neural processes. In all three experiments there is significant support for our linear regression’s y-intercept term being larger than zero. This is predicted by the Flexible Capacity model and indicative of shared resources being re-deployed according to changed task demands. By contrast, the Multiplicative Workload model cannot explain this since structural bottlenecks cannot, by definition, adapt. Our results extend Kahneman’s conception of neural processes competing with one another for resources beyond the ``decide-then-act” paradigm by providing evidence for the same type of interference effects during ``decide-while-acting” tasks. -- lines 626-640

With regards to Shiffrin & Schneider’s framework of controlled and automatic information processing, we have revised our manuscript to mention that controlled processes are those which are subject to our model, while automatic processes allow individuals to improve performance without exerting more effort, Importantly, the degree to which an individual can pay attention acts as a capacity limit on their ability to act. Not all neural processes require attention, Schneider and Shiffrin distinguish between automatic and controlled processes [59,61] and Kahneman distinguishes between System 1 and System 2 [32]. But processes that do require attention are subject to capacity limits and are prevented from occurring freely in parallel. These are the processes which are subject to constraints in our model. -- lines 619-625 and we now tie these observations into our discussion of the ecological impact that limited capacities have on the development of sensorimotor abilities, Considering that these bottlenecks occur when processes must compete for limited attention, it is important to note that controlled processes can be learned to the point where they become automatic processes and thereby reduce their demand for resources. Such learning reduces the instantaneous demands on shared resources during tasks and allows this limited capacity to be directed towards processing important stimuli and controlling increasingly complex responses [61]. -- lines 655-661

With regards to Navon and Gopher’s work on interpreting task difficulty in terms of resources, we have revised our manuscript to adopt their notion of demand composition to explain the differing effects of altered task demands within a multiple resource theory: Another question posed by shared resource models is the degree to which resources can be shared across all processes. Theories that posit the existence of multiple resources propose that different pools of resources are available to processes based on the processing stage, encoding, and modality of the process [76]. Task difficulty in these theories reflects both the degree to which individual processes exhaust their own resources and the amount of competition that exists for common resources [42]. Future work could aim to characterize the source and degree of difficulty in rapid motor behaviour tasks using performance operating characteristic curves [42,43,75]. -- lines 686-694

With regards to Sternberg’s body of work related to the performance of rapid movement sequences, we have revised our manuscript to comment that our steady-state rate is the kind of rate parameter that Sternberg et al. recommended for measuring participants’ performance of rapid movement sequences, such as keystrokes during typing,

We characterized participants’ performance during the overwhelmed phase by calculating their steady-state rate: the average target hit rate from the initial frame of the overwhelmed phase to the frame where the trial’s target creation rate peaked. The initial frame for this overwhelmed phase was then brought forward and the steady-state rate recalculated if a) doing so increased the participant’s steady-state rate or b) if the median rate difference minus the median absolute deviation was less than 0.1 Hz at any point in the preceding 5 s. This type of rate parameter is useful for characterizing performance for tasks involving ongoing movement and has been previously used in studying the latency and duration of keystroke sequences for typewriting tasks [66]. -- lines 176-185 and we now highlight that our model is consistent with Sternberg et al.’s proposed model of “two separate but concurrent and coordinated control processes for each hand” to explain the result that two-handed sequences are typed faster than one-hand sequences, especially for more skilled typists. Considering that these bottlenecks occur when processes must compete for limited attention, it is important to note that controlled processes can be learned to the point where they become automatic processes and thereby reduce their demand for resources. Such learning reduces the instantaneous demands on shared resources during tasks and allows this limited capacity to be directed towards processing important stimuli and controlling increasingly complex responses [61]. This kind of learning and the dedication of controlled and automatic processes to specific kinds of tasks may also support hierarchical control for motor actions. For example, there is evidence that skilled typists can type roughly twice as fast with two hands as they can with a single finger, but for student typists this is reversed [27]. A possible explanation for this type of effect is that each hand is controlled by separate hierarchical control processes which alternate between the controlled process of sequence planning and the automatic process of sequence execution [65]. Although we have not previously found evidence for the strategic alternation of hand movements in the OH family of tasks [40] there is clear scope for our model to be extended to account for the bimanual nature of many rapid motor behaviours tasks. -- lines 655-673

With regards to Wickens’s theory of multiple resources, we have revised our manuscript to draw explicit links between his description of data-limited and resource-limited tasks and our conception of easy and overwhelmed phases for both the OH family of tasks, Trials begin with objects created at a slow rate and with slow speeds. During this easy phase, the task is ``data-limited” and participants’ performance is limited by the input from the task [75]. Throughout the trial, however, objects are steadily created at a faster rate and with faster speeds, increasing perceptual, decision, and motor processing demands [74], ensuring that participants always finished a trial by being overwhelmed. Once they are overwhelmed, participants’ performance is resource-limited as they trade off speed, accuracy, and effort in order to maximize their global reward rate [20,75,56]. -- lines 128-136 and our simulations,

The simulation calculated the environment’s steadily increasing input rate throughout the trial according to (3), where t is the frame number, n is an additive noise factor drawn from the standard normal distribution N(0,1), and input(t) is the frame-specific input rate expressed in Hz. This ensured that every simulated trial started with an easy, data-limited phase and finished with an overwhelming, resource-limited phase. -- lines 343-348

Finally, we have revised our manuscript to draw upon the performance operating characteristics curve, used by Wickens, by Navon and Gopher, and by others, to suggest future research into the characteristics of these rapid motor behaviour tasks and the global vs. specialized nature of the brain’s central resources.

Another question posed by shared resource models is the degree to which resources can be shared across all processes. Theories that posit the existence of multiple resources propose that different pools of resources are available to processes based on the processing stage, encoding, and modality of the process [76]. Task difficulty in these theories reflects both the degree to which individual processes exhaust their own resources and the amount of competition that exists for common resources [42]. Future work could aim to characterize the source and degree of difficulty in rapid motor behaviour tasks using performance operating characteristic curves [42,43,75]. -- lines 686-694

In doing so, the following are a few issues the authors might address or at least consider: (a) is there a critical distinction between parallel and parallel-serial processing? For example, in your version in which two modules are modelled you suggest one might envision this as perceptual and decision processes occurring in one module while motor processes are resolved in a separate module. With this in mind, would resolving perceptual and decision uncertainly but not motor result in same/different outcomes as solving perceptual and motor uncertainty but not decision demands?

Based on feedback from another reviewer, we have named the parallel-serial processes that we discuss as pipelined processes, recalling the engineering and computing meanings of “pipeline.” The critical distinction between a pipelined process and straight parallel processes is that the former type of process consists of steps that must be completed sequentially (e.g., perception, decision, motor action), each of which may prove to be a limiting factor on system output, while the latter type of process is atomistic in its ability to be executed and completed. We have revised our manuscript to include this point:

We distinguish our systems’ pipelined processing from parallel-distributed processing because of the serial nature of these processes: perception must precede deliberation, which in turn must precede deliberate action, even if these processes do overlap and interact. These serial aspects open the door for any one aspect to become the limiting factor on the system’s output rate by becoming a bottleneck. -- lines 534-540

With this in mind, the effects of reducing a particular aspect of task difficulty depends on the role played by this aspect in limiting the system’s performance. Considering the OHA task in particular, if classifying target and distractors is the bottleneck process then we would expect to see a larger effect from reducing this perceptual/decision uncertainty (e.g., by indicating distractors with a distinctive colour) rather than reducing the uncertainty of motor actions (e.g., by providing larger paddles). (b) is serial-parallel processing different from parallel-distributed processing the latter of which might be more common nomenclature when this work is extended to consider neural implementation, an idea floated in parts of the paper.

As mentioned in our response above, we do see a critical difference between a pipeline process and a parallel-distributed process. Namely, the former consists of specifically identifiable steps, each of which might limit the output of the overall process. (c) I’m not convinced that the tasks used within the context of the KINARM are easy to distinguish based on the specific processing demands. While the “avoid” protocol likely loads on a decision process I am not so sure what the turbo version does. Again, I’m reminded of how carefully PIs selected experimental manipulations (i.e. conditions) to target specific processes to be clear what increased demand (see also additive factors methods). I’m not sure this is clear with these tasks, maybe it’s not that important right now. However, will it become important as this work is advanced?

We describe in our Methods section that [the turbo tasks] are speeded versions of the original tasks, with faster moving objects and smaller paddles (2 cm vs. 5 cm). -- lines 156-157 and that The original intent behind the task was to remove the ceiling effect that was specifically observed when elite athletes performed OH and OHA. -- lines 157-159

Within the context of this paper, Experiments 1 and 2 both compare tasks where the more difficult task adds the requirement to distinguish between targets and distractors. By contrast, in Experiment 3 the more difficult task reduces the time available per target and requires more precise motor actions due to the smaller size of paddle. We see the value of this Experiment as allowing us to demonstrate that the bottleneck effects of capacity limits can be seen for added difficulty in different task dimensions. We have revised our introduction to Experiment 3 to clarify this. Experiments 1 and 2 both assessed pairs of tasks where the additional difficulty was a result of the distractor objects, thus the dimension of increased task difficulty was the same for both experiments. In Experiment 3, we assessed our models against the performance of participants who performed both OH and TOH. In this pair of tasks, the increased difficulty occurs because the participant must use substantially smaller paddles to hit faster moving targets. This allows us to investigate whether the evidence found for limited capacity, pipeline processing generalizes beyond cases where an arbitrary decision rule is added. -- lines 506-513 (d) Is a single shared and flexible use resource the only capacity model to consider? I seem to recall that there was some thought that resources might be process specific. For example, I believe Wickens and others forwarded a multiple resource account some time ago. You are correct that a model of a single shared resource is not the only capacity model to consider. We have revised our manuscript to explicitly mention the multiple resource theory put forward by Wickens (and others) and we suggest the use of performance operating characteristics curves to address this question in future research.

Another question posed by shared resource models is the degree to which resources can be shared across all processes. Theories that posit the existence of multiple resources propose that different pools of resources are available to processes based on the processing stage, encoding, and modality of the process [76]. Task difficulty in these theories reflects both the degree to which individual processes exhaust their own resources and the amount of competition that exists for common resources [42]. Future work could aim to characterize the source and degree of difficulty in rapid motor behaviour tasks using performance operating characteristic curves [42,43,75]. -- lines 686-694

3. Reviewer 2

This paper evaluates how cognitive (i.e., attentional resources) are allocated across tasks (easy vs hard), comparing two different types of performers (high vs low). They found that behavior respects a Flexible Capacity model, in which sources can be allocated flexibly across different (motor, perceptual) modules depending on the demands of the task. This paper was nicely contextualized, the results were clear, and the conclusions were appropriate; I only have two minor suggestions for the authors to consider discussing.

Thank you for this high-level summary, we are pleased that you found it easy to follow our arguments. Thank you also for your two suggestions, they were helpful in pinpointing places where our logic was not fully presented for the reader. 1) The critical result that differentiated the multiplicative vs flexible capacity model was that the (inferred) intercept of the scatter plot was none-0 (Figure 5A). However, this seems to make a radical prediction that there would be, in theory, individuals who exert greater output in task 2 (the harder task) compared to task 1, if we follow the line close to (0, 0). Why were there no individuals here? Are behavioral manipulations that can reveal such behavior?

You are correct that a straightforward interpretation of our Flexible Capacity model would imply that there are individuals who reliably score better on more difficult tasks. We have revised our explanation of the model’s prediction to clarify that only the portion of the regression line that is to the right of the unity line is valid (much in the same way that the Additive Workload model does not actually predict that some participants will produce negative output for more difficult tasks).

Note that the predictions made by the Additive Workload and Flexible Capacity models are only valid to the right of the unity line in quadrant I: neither model predicts output that is either negative or increased for the more difficult task.-- lines 395-398 2a) The authors argue that this is not motivation, but I don’t quite follow the reasoning on page 10 “if motivation played a significant role, then we would expect to see some participants exhibit substantial increases or decreases in hitting rate during their overwhelmed phase?” Have the authors shown that there are no substantial fluctuations in the overwhelmed phase?

We interpret the steady-state rate that we measure for participants as a measure of their peak performance and discount the potentially confounding role played by motivation. We think this is appropriate because there are three places where motivation could affect the steady-state rates that we measure: 1. Within a trial, fluctuations in motivation would result in significant deviations in steady-state rates.

2. Between trials, fluctuations in motivation would result in significant differences between steady-state rates.

3. As a global parameter, if a participant’s level of motivation is a consistent factor in determining the steady-state rate then we consider it to be a part of that participant’s underlying ability.

We have considered these three places and describe our reasoning in the paragraph you highlight:

1. We assessed the fluctuations of steady-state rates within the overwhelmed phase of trials and found no evidence of substantial changes that might be explained by within-trial changes in motivation.

2. We assessed differences in steady-state rates for participants who performed multiple trials of a single task and found that these differences are quite small, providing no evidence for either changed motivation levels or learning effects between trials.

3. If a participant’s level of motivation consistently limits their performance, then we consider it as much a part of their underlying ability as a maximum hand speed. We have revised the paragraph in question to clarify this reasoning: We interpret a participant’s steady-state rate for a trial as representing their peak performance and underlying sensorimotor abilities. Although it is difficult to untangle the effects of factors such as motivation, we believe that this interpretation is justified for three reasons. First, if motivation levels determined steady-state rates within a trial then we would expect to see some participants either substantially increase or substantially decrease their target hit rate during the overwhelmed phase as their motivation fluctuated, but this was not observed. Second, if motivation levels determined steady-state rates between trials then we would expect some participants to exhibit a wide range of steady-state rates across trials. Instead, all four tasks have been validated as evoking consistent performance levels and steady-state rates between tests.

Third, if motivation levels determine steady-state rates between participants, this role seems consistent within and between trials for single participants. We would therefore consider this kind of effect as a part of the participant’s underlying ability to perform rapid motor behaviour tasks. -- lines 186-200

2b) Is it possible that the steady-state reflects the level of motivation participants set for both easy and hard tasks? That is, if you incentivized participants with more money, would the participants remain at the same steady state (more persistent trait-like quality), or could financial incentives motivate participants to modulate their steady-state rate (incidental state-like quality)? If the latter, then how do the authors conceptualize the possibility of this steady-state reflecting a self-selected ‘motivational’ threshold rather than a true sensorimotor/attentional ‘information bottleneck’? It is possible that if our rapid motor behaviour tasks remained in the “easy” range for the entire duration that motivation levels might play a role. Such tasks are data-limited and system output (or participant performance) is limited by the input available from the environment and there is no incentive to apply a participant’s maximum resources (motivation or otherwise) to the task. Kahneman noted that for such tasks, participants reliably do not exert all their potential effort even though this added effort can be brought forward with appropriate incentives.

By contrast, we studied the overwhelmed phase of rapid motor behaviour tasks where performance is resource-limited, and all resources must be applied to achieve and maintain peak performance. Since this portion of the tasks call for the usage of all available resources and since we saw no evidence for effects of motivation on this peak performance, we do not believe that the steady-state rates we measure reflect the participants’ underlying motivation levels.

Altogether, I thoroughly enjoyed reading the paper, and am envious of their sample size!

Thank you!

## References

[B1] Adkins T, Lewis R, Lee T (2022) Heuristics contribute to sensorimotor decision-making under risk. Psychon Bull Rev 29:145–158. 10.3758/s13423-021-01986-x 34508307

[B2] Amrhein V, Greenland S, McShane B (2019) Scientists rise up against statistical significance. Nature 567:305–307. 10.1038/d41586-019-00857-9 30894741

[B3] Balci F, Simen P, Niyogi R, Saxe A, Hughes JA, Holmes P, Cohen JD (2011) Acquisition of decision making criteria: reward rate ultimately beats accuracy. Atten Percept Psychophys 73:640–657. 10.3758/s13414-010-0049-7 21264716 PMC3383845

[B4] Barany DA, Gómez-Granados A, Schrayer M, Cutts SA, Singh T (2020) Perceptual decisions about object shape bias visuomotor coordination during rapid interception movements. J Neurophysiol 123:2235–2248. 10.1152/jn.00098.2020 32374224

[B5] Bogacz R, Hu PT, Holmes PJ, Cohen JD (2010) Do humans produce the speed–accuracy trade-off that maximizes reward rate? Q J Exp Psychol (Hove) 63:863–891. 10.1080/17470210903091643 19746300 PMC2908414

[B6] Bourke TC, Lowrey CR, Dukelow SP, Bagg SD, Norman KE, Scott SH (2016) A robot-based behavioural task to quantify impairments in rapid motor decisions and actions after stroke. J Neuroeng Rehabil 13:91. 10.1186/s12984-016-0201-2 27724945 PMC5057404

[B7] Brandstätter E, Körner C (2014) Attention in risky choice. Acta Psychol (Amst) 152:166–176. 10.1016/j.actpsy.2014.08.008 25226548

[B8] Broadbent DE (1957) A mechanical model for human attention and immediate memory. Psychol Rev 64:205–215. 10.1037/h0047313 13441856

[B9] Broadbent DE (1971) Decision and stress. London: Academic Press Inc. (London) Ltd.

[B10] Carroll TJ, McNamee D, Ingram JN, Wolpert DM (2019) Rapid visuomotor responses reflect value-based decisions. J Neurosci 39:3906–3920. 10.1523/JNEUROSCI.1934-18.2019 30850511 PMC6520503

[B11] Cisek P (2007) Cortical mechanisms of action selection: the affordance competition hypothesis. Philos Trans R Soc Lond B Biol Sci 362:1585–1599. 10.1098/rstb.2007.2054 17428779 PMC2440773

[B12] Cisek P (2019) Resynthesizing behavior through phylogenetic refinement. Atten Percept Psychophys 81:2265–2287. 10.3758/s13414-019-01760-1 31161495 PMC6848052

[B13] Cisek P, Kalaska JF (2010) Neural mechanisms for interacting with a world full of action choices. Annu Rev Neurosci 33:269–298. 10.1146/annurev.neuro.051508.135409 20345247

[B14] Cisek P, Pastor-Bernier A (2014) On the challenges and mechanisms of embodied decisions. Philos Trans R Soc Lond B Biol Sci 369:20130479. 10.1098/rstb.2013.0479 25267821 PMC4186232

[B15] Cisek P, Thura D (2018) Neural circuits for action selection. In: Reach-to-grasp behavior: brain, behavior, and modelling across the life span, pp 91–118. New York: Routledge.

[B16] Cos I, Duque J, Cisek P (2014) Rapid prediction of biomechanical costs during action decisions. J Neurophysiol 112:1256–1266. 10.1152/jn.00147.2014 24899673

[B17] Deutsch J, Deutsch D (1963) Attention: some theoretical considerations. Psychol Rev 70:80–90. 10.1037/h0039515 14027390

[B18] Diamond JS, Wolpert DM, Flanagan JR (2017) Rapid target foraging with reach or gaze: the hand looks further ahead than the eye. PLoS Comput Biol 13:e1005504. 10.1371/journal.pcbi.1005504 28683138 PMC5500014

[B19] Enachescu V, Schrater P, Schaal S, Christopoulos V (2021) Action planning and control under uncertainty emerge through a desirability-driven competition between parallel encoding motor plans. PLoS Comput Biol 17:e1009429. 10.1371/journal.pcbi.1009429 34597294 PMC8513832

[B20] Fitts PM (1954) The information capacity of the human motor system in controlling the amplitude of movement. J Exp Psychol 47:381–391. 10.1037/h0055392 13174710

[B21] Fooken J, Spering M (2020) Eye movements as a readout of sensorimotor decision processes. J Neurophysiol 123:1439–1447. 10.1152/jn.00622.2019 32159423 PMC7191514

[B22] Franconeri SL, Alvarez GA, Enns JT (2007) How many locations can be selected at once? J Exp Psychol Hum Percept Perform 33:1003–1012. 10.1037/0096-1523.33.5.1003 17924803

[B23] Franconeri SL, Alvarez GA, Cavanagh P (2013) Flexible cognitive resources: competitive content maps for attention and memory. Trends Cogn Sci 17:134–141. 10.1016/j.tics.2013.01.010 23428935 PMC5047276

[B24] Gallivan JP, Chapman CS, Wood DK, Milne JL, Ansari D, Culham JC, Goodale MA (2011) One to four, and nothing more: nonconscious parallel individuation of objects during action planning. Psychol Sci 22:803–811. 10.1177/0956797611408733 21562312

[B25] Gallivan JP, Barton KS, Chapman CS, Wolpert DM, Randall Flanagan J (2015) Action plan co-optimization reveals the parallel encoding of competing reach movements. Nat Commun 6:7428. 10.1038/ncomms8428 26130029 PMC4502063

[B26] Gallivan JP, Chapman CS, Wolpert DM, Flanagan JR (2018) Decision-making in sensorimotor control. Nat Rev Neurosci 19:519–534. 10.1038/s41583-018-0045-9 30089888 PMC6107066

[B27] Gentner DR (1983) Keystroke timing in transcription typing. In: Cognitive aspects of skilled typewriting (Cooper W, ed.), Chapter 5, pp 95–120. New York: Springer New York Inc. 10.1007/978-1-4612-5470-6

[B28] Haith AM, Huberdeau DM, Krakauer JW (2015) Hedging your bets: intermediate movements as optimal behavior in the context of an incomplete decision. PLoS Comput Biol 11:e1004171. 10.1371/journal.pcbi.1004171 25821964 PMC4379031

[B29] Hommel B, Chapman CS, Cisek P, Neyedli HF, Song J-H, Welsh TN (2019) No one knows what attention is. Atten Percept Psychophys 81:2288–2303. 10.3758/s13414-019-01846-w 31489566 PMC6848248

[B30] Jarvstad A, Hahn U, Rushton SK, Warren PA (2013) Perceptuo-motor, cognitive, and description-based decision-making seem equally good. Proc Natl Acad Sci U S A 110:16271–16276. 10.1073/pnas.1300239110 24048030 PMC3791786

[B31] Kahneman D (1973) Attention and effort. Eaglewood Cliffs: Prentice-Hall Inc.

[B32] Kahneman D (2011) Thinking, fast and slow. London: Penguin Books.

[B33] Kruschke JK (2015) Doing Bayesian data analysis: a tutorial with R, JAGS, and Stan, Ed 2. New York: Academic Press.

[B34] Kruschke JK, Liddell TM (2018) The Bayesian new statistics: hypothesis testing, estimation, meta-analysis, and power analysis from a Bayesian perspective. Psychon Bull Rev 25:178–206. 10.3758/s13423-016-1221-4 28176294

[B35] Künstler ECS, Finke K, Günther A, Klingner C, Witte O, Bublak P (2018) Motor-cognitive dual-task performance: effects of a concurrent motor task on distinct components of visual processing capacity. Psychol Res 82:177–185. 10.1007/s00426-017-0951-x 29196834 PMC5816117

[B36] Lepora NF, Pezzulo G (2015) Embodied choice: how action influences perceptual decision making. PLoS Comput Biol 11:e1004110. 10.1371/journal.pcbi.1004110 25849349 PMC4388485

[B37] Mang CS, Whitten TA, Cosh MS, Scott SH, Wiley JP, Debert CT, Dukelow SP, Benson BW (2018) Test-retest reliability of the KINARM end-point robot for assessment of sensory, motor and neurocognitive function in young adult athletes. PLoS One 13:e0196205. 10.1371/journal.pone.0196205 29689075 PMC5915777

[B38] Michalski J, Green AM, Cisek P (2020) Reaching decisions during ongoing movements. J Neurophysiol 123:1090–1102. 10.1152/jn.00613.2019 32049585 PMC7099481

[B39] Morasso P (1981) Spatial control of arm movements. Exp Brain Res 42:223–227. 10.1007/BF00236911 7262217

[B40] Moulton RH, Rudie K, Dukelow SP, Scott SH (2022) Quantitatively assessing aging effects in rapid motor behaviours: a cross-sectional study. J Neuroeng Rehab 19:82. 10.1186/s12984-022-01035-1 PMC9327262 35883179

[B41] Nagengast AJ, Braun DA, Wolpert DM (2011) Risk-sensitivity and the mean-variance trade-off: decision making in sensorimotor control. Proc Biol Sci 278:2325–2332. 10.1098/rspb.2010.2518 21208966 PMC3119020

[B42] Navon D, Gopher D (1978) Interpretations of task difficulty in terms of resources: efficiency, load, demand and cost composition. Technical report. Haifa: Israel Institute of Technology.

[B43] Navon D, Gopher D (1979) On the economy of the human-processing system. Psychol Rev 86:214–255. 10.1037/0033-295X.86.3.214

[B44] Navon D, Miller J (2002) Queuing or sharing? A critical evaluation of the single-bottleneck notion. Cogn Psychol 44:193–251. 10.1006/cogp.2001.0767 11971632

[B45] Neyedli HF, LeBlanc KA (2017) The role of consistent context in rapid movement planning: suboptimal endpoint adjustment to changing rewards. J Mot Behav 49:697–707. 10.1080/00222895.2016.1271296 28481692

[B46] Neyedli HF, Welsh TN (2013) Optimal weighting of costs and probabilities in a risky motor decision-making task requires experience. J Exp Psychol Hum Percept Perform 39:638–645. 10.1037/a0030518 23163791

[B47] Neyedli HF, Welsh TN (2015) Experience and net worth affects optimality in a motor decision task. Motor Control 19:75–89. 10.1123/mc.2013-0024 25029662

[B48] Nook EC, Lindquist KA, Zaki J (2015) A new look at emotion perception: concepts speed and shape facial emotion recognition. Emotion 15:569–578. 10.1037/a0039166 25938612

[B49] Nuzzo R (2014) Statistical errors: p values, the “gold standard” of statistical validity, are not as reliable as many scientists assume. Nature 506:150–152. 24522584 10.1038/506150a

[B50] Orquin JL, Mueller Loose S (2013) Attention and choice: a review on eye movements in decision making. Acta Psychol (Amst) 144:190–206. 10.1016/j.actpsy.2013.06.003 23845447

[B51] Passingham RE (1996) Attention to action. Philos Trans R Soc Lond B Biol Sci 351:1473–1479. 10.1098/rstb.1996.0132 8941959

[B52] Pezzulo G, Ognibene D (2012) Proactive action preparation: seeing action preparation as a continuous and proactive process. Motor Control 16:386–424. 10.1123/mcj.16.3.386 22643383

[B53] Raßbach P, Grießbach E, Cañal-Bruland R, Herbort O (2021) Deciding while moving: cognitive interference biases value-based decisions. Acta Psychol (Amst) 221:103449. 10.1016/j.actpsy.2021.103449 34801882

[B54] Ratcliff R, McKoon G (2008) The diffusion decision model: theory and data for two-choice decision tasks. Neural Comput 20:873–922. 10.1162/neco.2008.12-06-420 18085991 PMC2474742

[B55] Redish AD (2013) The mind within the brain: how we make decisions and how those decisions go wrong. New York: Oxford University Press.

[B56] Reynaud AJ, Saleri Lunazzi C, Thura D (2020) Humans sacrifice decision-making for action execution when a demanding control of movement is required. J Neurophysiol 124:497–509. 10.1152/jn.00220.2020 32639900

[B57] Scherbaum S, Gottschalk C, Dshemuchadse M, Fischer R (2015) Action dynamics in multitasking: the impact of additional task factors on the execution of the prioritized motor movement. Front Psychol 6:934–938. 10.3389/fpsyg.2015.00934 26217267 PMC4491597

[B58] Schmidt RA, Wrisberg CA (2004) Motor learning and performance, Ed 3. Champaign: Human Kinetics.

[B59] Schneider W, Shiffrin RM (1977) Controlled and automatic human information processing: I. Detection, search, and attention. Psychol Rev 84:1–66. 10.1037/0033-295X.84.1.1

[B60] Scott SH (2016) A functional taxonomy of bottom-up sensory feedback processing for motor actions. Trends Neurosci 39:512–526. 10.1016/j.tins.2016.06.001 27378546

[B61] Shiffrin RM, Schneider W (1977) Controlled and automatic human information processing: II. Perceptual learning, automatic attending and a general theory. Psychol Rev 84:127–190. 10.1037/0033-295X.84.2.127

[B62] Simmatis LE, Early S, Moore KD, Appaqaq S, Scott SH (2020) Statistical measures of motor, sensory and cognitive performance across repeated robot-based testing. J Neuroeng Rehabil 17:86. 10.1186/s12984-020-00713-2 32615979 PMC7331240

[B63] Simons DJ, Chabris CF (1999) Gorillas in our midst: sustained inattentional blindness for dynamic events. Perception 28:1059–1074. 10.1068/p281059 10694957

[B64] Singh K, Scott SH (2003) A motor learning strategy reflects neural circuitry for limb control. Nat Neurosci 6:399–403. 10.1038/nn1026 12627165

[B65] Sternberg S, Monsell S, Knoll RL, Wright CE (1978) The latency and duration of rapid movement sequences: comparisons of speech and typewriting. In: Information processing in motor control and learning (Stelmach G, ed.), Chapter 6, pp 117–152. New York: Academic Press.

[B66] Sternberg S, Knoll RL, Turock DL (1990) Hierarchical control in the execution of action sequences: tests of two invariance properties. In: Attention and performance XIII (Jeannerod M, ed.), Chapter 1, pp 3–55. Hillsdale: Erlbaum. 10.4324/9780203772010

[B67] Thorpe S, Fize D, Marlot C (1996) Speed of processing in the human visual system. Nature 381:520–522. 10.1038/381520a0 8632824

[B68] Thura D (2020) Decision urgency invigorates movement in humans. Behav Brain Res 382:112477. 10.1016/j.bbr.2020.112477 31926934

[B69] Thura D, Cisek P (2014) Deliberation and commitment in the premotor and primary motor cortex during dynamic decision making. Neuron 81:1401–1416. 10.1016/j.neuron.2014.01.031 24656257

[B70] Treisman AM (1969) Strategies and models of selective attention. Psychol Rev 76:282–299. 10.1037/h0027242 4893203

[B71] Trommershäuser J, Maloney LT, Landy MS (2008) Decision making, movement planning and statistical decision theory. Trends Cogn Sci 12:291–297. 10.1016/j.tics.2008.04.010 18614390 PMC2678412

[B72] Tyryshkin K, Coderre AM, Glasgow JI, Herter TM, Bagg SD, Dukelow SP, Scott SH (2014) A robotic object hitting task to quantify sensorimotor impairments in participants with stroke. J Neuroeng Rehabil 11:47–12. 10.1186/1743-0003-11-47 24693877 PMC3992166

[B73] Ulbrich P, Gail A (2021) The cone method: inferring decision times from single-trial 3D movement trajectories in choice behavior. Behav Res Methods 53:2456–2472. 10.3758/s13428-021-01579-5 33852130 PMC8613081

[B74] Vidal F, Meckler C, Hasbroucq T (2015) Basics for sensorimotor information processing: some implications for learning. Front Psychol 6:33–14. 10.3389/fpsyg.2015.00033 25762944 PMC4329794

[B75] Wickens CD (1981) Processing resources in attention, dual task performance, and workload assessment. Technical report. Arlington: Office of Naval Research, Engineering Psychology Program.

[B76] Wickens CD (2008) Multiple resources and mental workload. Hum Factors 50:449–455. 10.1518/001872008X288394 18689052

[B77] Wolpert DM, Diedrichsen J, Flanagan JR (2011) Principles of sensorimotor learning. Nat Rev Neurosci 12:739–751. 10.1038/nrn3112 22033537

[B78] Wu SW, Delgado MR, Maloney LT (2009) Economic decision-making compared with an equivalent motor task. Proc Natl Acad Sci U S A 106:6088–6093. 10.1073/pnas.0900102106 19332799 PMC2669401

